# A bifunctional ER-targeting fluorescent probe for the detection of SO_2_ and polarity in living cell and animals

**DOI:** 10.1039/d6sc05911b

**Published:** 2026-10-14

**Authors:** Hao Wu, Yue Sun, Yuan Wang, Jing Zhang, Wei-Na Wu, Zhi-Hong Xu, Tony D. James

**Affiliations:** a Translational Research on Cell and Gene Therapy for Higher Education Institutions for Shaanxi key Laboratory of Higher Education Institutions, Xi'an Medical University Xi'an Shaanxi 710021 PR China jingzhang@xiyi.edu.cn; b College of Chemistry and Chemical Engineering, Henan Key Laboratory of Coal Green Conversion, Henan Polytechnic University Jiaozuo 454000 PR China wangyuan08@hpu.edu.cn; c Key Laboratory of Chemo/Biosensing and Detection of Xuchang, Key Laboratory of Micro-Nano Materials for Energy Storage and Conversion of Henan Province, Henan Joint International Research Laboratory of Nanomaterials for Energy and Catalysis, College of Chemical and Materials Engineering, Xuchang University 461000 P.R. China xuzhihong1980@xcu.edu.cn; d Department of Chemistry, University of Bath Bath BA2 7AY UK t.d.james@bath.ac.uk; e School of Chemistry and Chemical Engineering, Henan Normal University Xinxiang. 453007 PR China

## Abstract

SO_2_ and solvent polarity are critical for physiological homeostasis, and their dysregulation is implicated in various diseases, including cancer. In this study, we report a novel intramolecular charge transfer (ICT), reaction based two channel ratiometric fluorescent probe, (*E*)-2-cyano-*N*-(3-((4-methylphenyl)sulfonamido)propyl)-3-(4-oxo-7,8,11,12-tetrahydro-4*H*,6*H*, 10*H*-pyrido[3,2,1-*ij*]thieno[2′,3':4,5]pyrano[2,3-*f*]quinolin-2-yl)acrylamide (CTAT), for the simultaneous detection of SO_2_ and microenvironmental polarity *via* two fluorescence channels. CTAT exhibits reaction based two channel ratiometric responses (*λ*_ex_ = 410 nm, *λ*_em_ = 490 nm; *λ*_ex_ = 510 nm, *λ*_em_ = 660 nm) and distinct colorimetric fluorescence changes upon reaction with SO_2_, with a low detection limit of 0.144 µM. CTAT also sensitively reports solvent polarity: in 1,4-dioxane/PBS mixtures where it displays an emission peak at 660 nm, and its maximum fluorescence intensity (*F*_max_) correlates linearly with the polarity parameter Δ*f* across the tested range. CTAT exhibits low phototoxicity and good biocompatibility, as well as strong endoplasmic reticulum targeting and as such was successfully applied to image endogenous and exogenous SO_2_ derivatives and polarity changes in HepG2 and L-02 living cells, zebrafish, and tissue samples. This research provides a new strategy and tool for diagnosing polarity-related diseases and for detecting SO_2_ in real environments.

## Abstract 2

CTAT is a dual-excitation, ER-targeted fluorescent probe enabling crosstalk-free detection of HSO_3_^−^ and polarity, exhibiting simultaneous monitoring in live cells and zebrafish and high sensitivity and selectivity in disease models.

## Graphical abstract

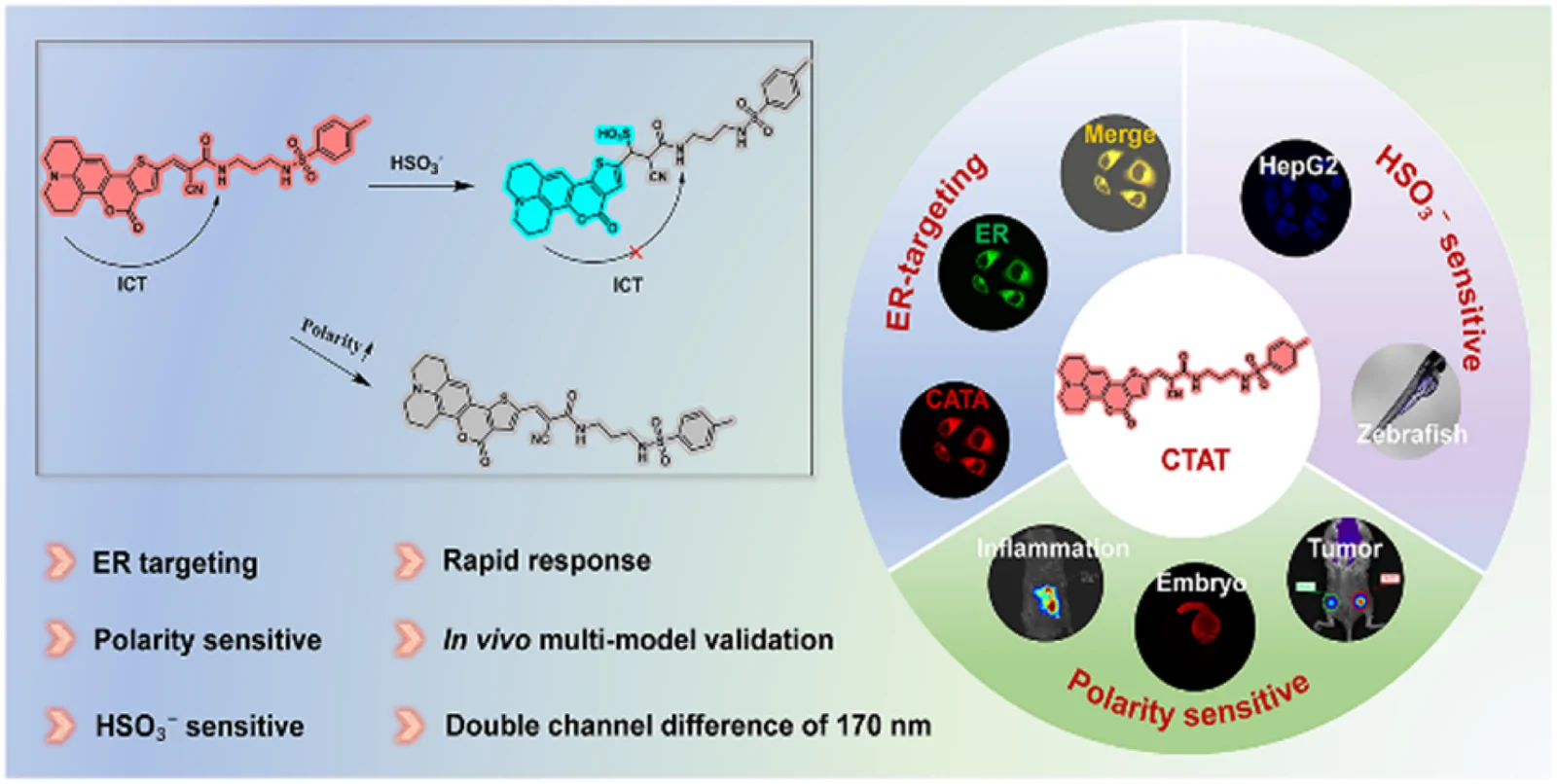

## Introduction

Sulfur dioxide (SO_2_) is an important gaseous signaling molecule in biological systems, following the discovery of other gaseous signaling molecules such as nitric oxide (NO), carbon monoxide (CO), and hydrogen sulfide (H_2_S), and can be readily hydrated in aqueous environments to form sulfite and bisulfite species (SO_3_^2−^/HSO^3−^).^1–3^ Furthermore, SO_2_ and its derivatives within living cells are crucial in physiological and pathological processes,^4,5^ particularly in regulating cellular stress responses and maintaining ER homeostasis.^6,7^ Dysregulation of SO_2_ metabolism or signaling has been associated with ER stress, which contributes to diseases such as liver injury, lung injury, myocardial infarction, neurodegenerative diseases, cancer, and inflammatory diseases.^8–11^ The ER is a central organelle involved in protein folding, redox regulation, calcium storage, and lipid biosynthesis. These functions are highly sensitive to changes in the local physicochemical microenvironment. In particular, alterations in ER polarity may reflect changes in membrane composition and lipid organization, and may serve as an indicator of ER microenvironmental perturbation.^12–17^ Therefore, simultaneous monitoring of SO_2_ derivatives and polarity in the ER could provide complementary information for investigating the relationship between sulfur-related signaling and ER homeostasis. Unlike analytical methods such as electrochemistry,^18^ electrophoresis,^19^ and chromatography,^20^ fluorescence probes are emerging as major research tools in the fields of chemistry, biology, and medicine due to their high sensitivity, selectivity, non-invasiveness, excellent spatiotemporal resolution, and real-time monitoring capabilities.^21,22^

Over the past several years, a variety of polarity probes and SO_2_ probes have been developed. Nevertheless, the majority of the probes reported previously only respond to one type of chemical analyte, while fluorescent probes capable of simultaneously monitoring both polarity and SO_2_ are currently limited.^10,23–25^ In particular, ratiometric fluorescent probes offer reliable and visualizable detection of SO_2_, mitigating the impact of photobleaching, probe concentration, and measurement conditions.^3^ However, conventional single-excitation probes suffer from signal distortion and limited applications. Dual-excitation probes, with their two independent signal sets, better address complex environmental changes, reduce background interference, and minimize signal distortion.^26^ Despite these advantages, reaction based two channel ratiometric fluorescent probes for SO_2_ detection are scarce.^27^ Furthermore, while existing SO_2_ fluorescent probes often target mitochondria and lysosomes, probes targeting the endoplasmic reticulum are limited.^1,4,27–29^ Consequently, the construction of a reaction-based two-channel ratiometric fluorescent probe capable of targeting the ER and simultaneously detecting SO_2_ derivatives and polarity is highly desirable, not only for fluorescence imaging but also for exploring ER-associated microenvironmental changes.^30–32^

With this research, we selected coumarin as the fluorescent donor, cyanoacetamide as an acceptor, and introduced a thiophene group to improve the fluorescence properties, resulting in a new bifunctional endoplasmic reticulum-targeted ratiometric fluorescent probe CTAT based on an intramolecular charge transfer (ICT) mechanism, for detecting SO_2_ and polarity in living systems (Scheme 1). Not unexpectedly, CTAT exhibited a red emission at 660 nm when excited at 510 nm, which was selectively quenched by SO_2_ (as HSO_3_^−^, a common SO_2_ source). While, a new emission at 490 nm was generated with an excitation wavelength of 410 nm, thereby realizing reaction based two channel ratiometric fluorescent detection of SO_2_. In addition, both the absorption and emission intensities of CTAT decreased with increasing solvent polarity, accompanied by slight red shifts. This distinct ratiometric signal was used to enable the monitoring of exogenous/endogenous SO_2_ and polarity *in vitro* and *in vivo*.

**Scheme 1:**
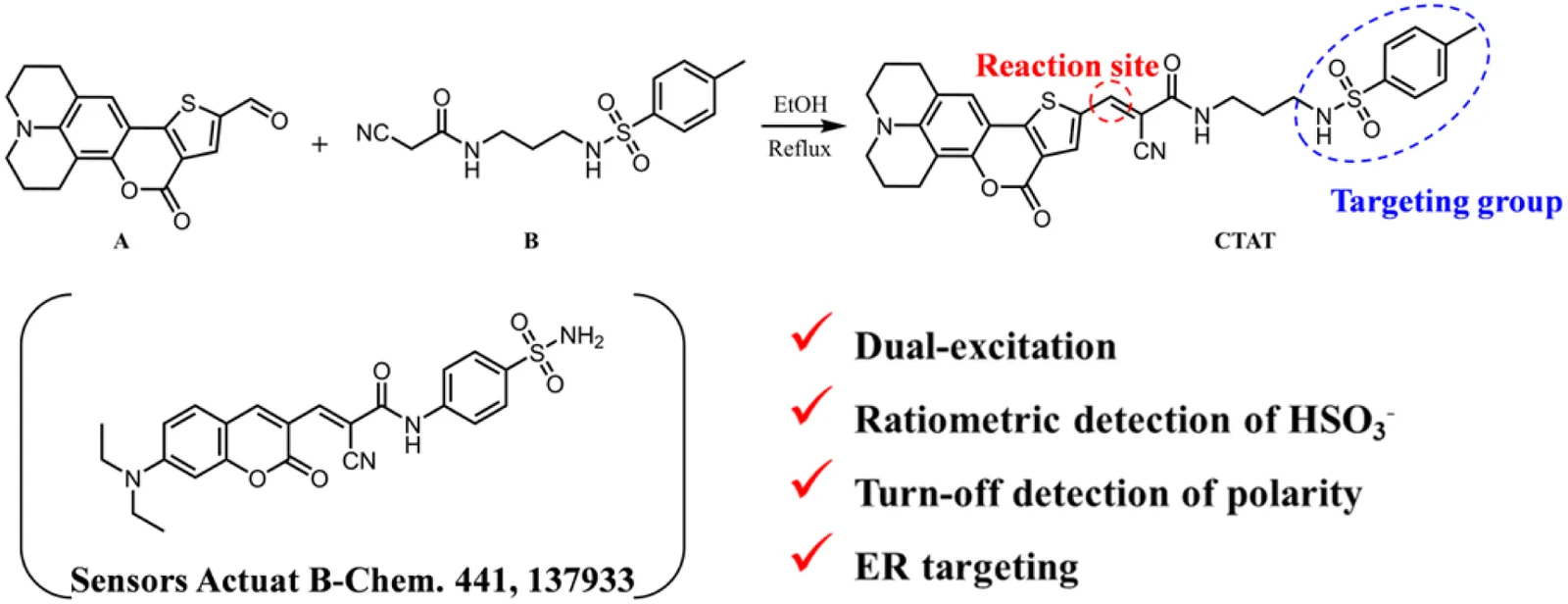
Synthetic route for probe CTAT.

## Results and discussion

### Optical response to polarity by probe CTAT

First, the photophysical properties of CTAT in various solvents were determined (Fig. 1 and S3). As shown in Fig. 1a and S3, with an increase of solvent polarity, the absorption (510 nm) and emission (660 nm) peaks of the probe undergo obvious decreases. To further investigate the effect of polarity on CTAT, UV-Vis absorption and fluorescence spectra were recorded for the probe in PBS/1,4-dioxane mixtures with different polarity. Fig. 1b indicates that as the PBS content increases from 10% to 60%, the maximum emission peak not only decreases significantly but also exhibits a red shift (approximately 40 nm), while the maximum absorption peak also displays a slight red shift of about 10 nm. Additionally, as the solution's polarity shifts from 0.18 (80% water) to 0.30 (20% water), the fluorescence intensity (*F*_max_) of CTAT exhibits a strong linear correlation with Δ*f* across the polarity range (Fig. 1c). These results confirm that the CTAT probe is extremely sensitive to changes in polarity and as such is expected to serve as a reliable tool for detecting the polarity of aqueous solutions.

**Fig. 1:**
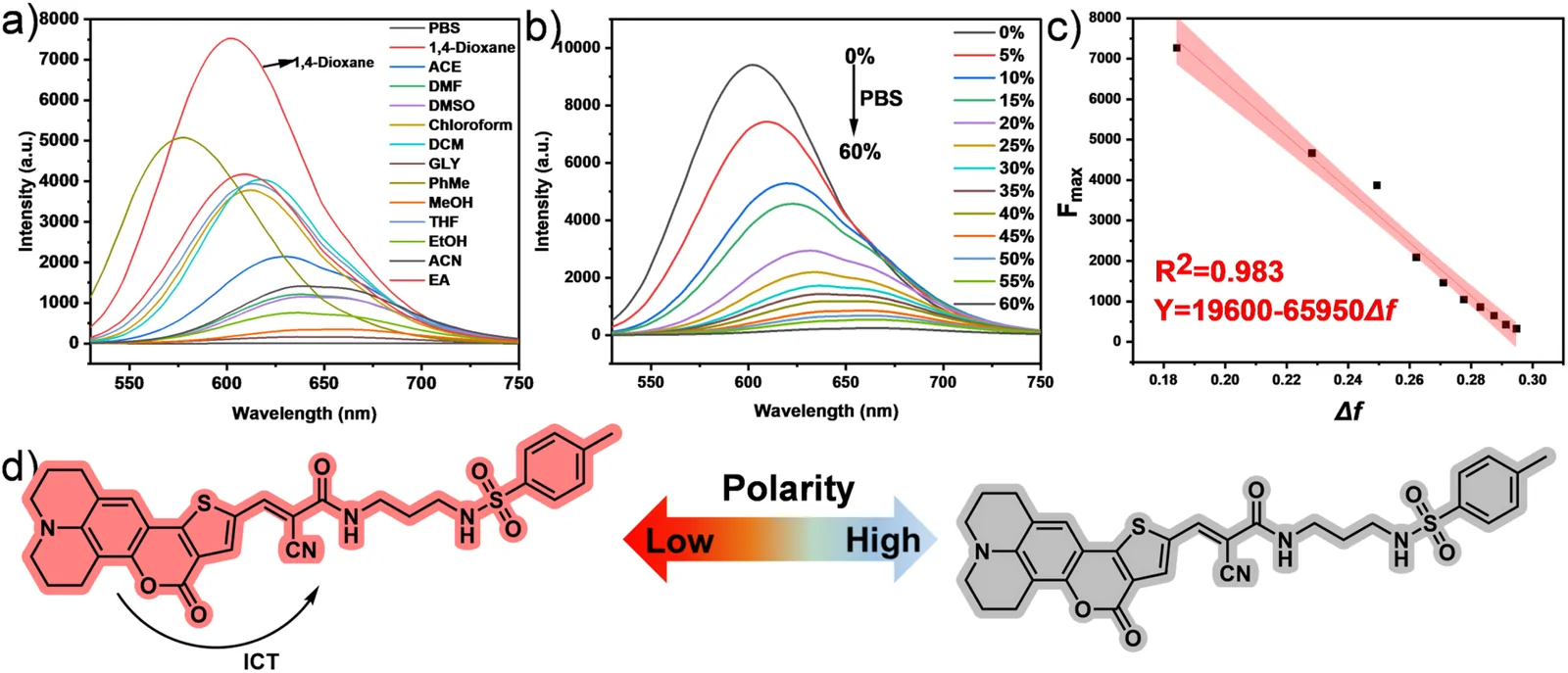
(a) Fluorescence spectra of probe CTAT (10 µM) in various solvents, (b) fluorescence spectra of CTAT (10 µM) in PBS/1,4-dioxane solvent mixtures with different volume ratios under excitation at 510 nm. (c) Linear relationship between the fluorescence intensity of CTAT at *F*_660_ and the solvent parameter Δ*f*. (d) The proposed mechanism of CTAT with polarity.

### Spectral response of probe CTAT to HSO_3_^−^

Sensitivity is an important aspect for the detection by photochemical sensors. We examined the UV-Vis absorption and fluorescence spectra of probe CTAT (10 µM) with and without HSO_3_^−^ in EtOH/PBS (3 : 7, v/v, pH = 7.4), and conducted concentration-dependent titration experiments. As depicted in Fig. S4a, CTAT exhibited a strong absorption peak at 510 nm. Upon the addition of HSO_3_^−^, the absorption band of CTAT centered at 510 nm was blue-shifted to 410 nm accompanied by a noticeable color change from pink to colorless, whereas the absorbance and color of the CTAT solution was not significantly affected by other tested analytes. These results indicate that CTAT was able to detect HSO_3_^−^ using the naked eye. Furthermore, when the concentration of HSO_3_^−^ increased from 0 µM to 100 µM, the absorbance intensity at 510 nm gradually decreased, and the absorbance intensity at 410 nm gradually increased (Fig. S4b). A good linear relationship (*R*^2^ = 0.996) was obtained between the concentration of HSO_3_^−^ (20–80 µM) and the absorbance intensity ratio of *A*_410_/*A*_510_, indicating that CTAT can quantitatively detect HSO_3_^−^ by colorimetric analysis (Fig. S4c).

Thereupon, we explored the fluorescence spectra of CTAT on reaction with HSO_3_^−^ with an excitation wavelength at 410 nm and 510 nm. As shown in Fig. 1b, CTAT emitted red fluorescence, and a strong emission peak at 660 nm (*λ*_ex_ = 510 nm). Significantly, when different species were added (Ag^+^, Al^3+^, Mn^2+^, Mg^2+^, Zn^2+^, K^+^, Ca^2+^, Cd^2+^, Co^3+^, Cr^3+^, Cu^2+^, Hg^2+^, Fe^3+^, Pb^2+^, Na^+^, Ni^2+^, Cl^−^, ClO^−^, ClO_4_^−^, F^−^, Br^−^, H_2_PO_4_^−^, HPO_4_^2−^, HSO^4−^, SO_3_^2−^, SO_4_^2−^, OAc^−^, PO_4_^3−^, I^−^, S_2_O_5_^2−^, S_2_O_4_^2−^, S_2_O_3_^2−^, S^2−^, PPi, Hcy, Cys, GSH, ^1^O_2_, Ala, NO·, Leu, ROO^−^, HSO_3_^−^, 10 eq.) to the test solution, only the addition of HSO_3_^−^ resulted in the fluorescence intensity of CTAT at 660 nm to slowly decrease, the ICT effect was destroyed, and a new increasing blue fluorescence emission peak at 490 nm (*λ*_ex_ = 410 nm) emerged (Fig. S5), and the fluorescence color of the solution changed from orange to blue under a hand-held 365 nm lamp (Fig. 2a). Furthermore, we evaluated the anti-interference ability of the probe for HSO_3_^−^ detection using competitive experiments. The detection of HSO_3_^−^ by the probe was hardly affected when the above-mentioned individual analytes were added to a probe solution containing HSO_3_^−^ (Fig. 2b). These results indicated that the probe CTAT can specifically detect HSO_3_^−^ with negligible interference from other analytes. Moreover, a good linear relationship was established between the signal ratio (*F*_490_/*F*_660_) and HSO_3_^−^ concentration (0–80 µM) (*R*^2^ = 0.990). Based on the formula 3*δ*/*k*, the detection limit was calculated to be 0.144 µM (Fig. 2c), suggesting that CTAT could function as a reliable quantitative tool for probing HSO_3_^−^ derivatives *in vitro*. Additional spectroscopic experiments confirmed that the fluorescence responses of CTAT toward SO_2_ derivatives and polarity were largely independent, with negligible mutual interference (Fig. S6).

**Fig. 2:**
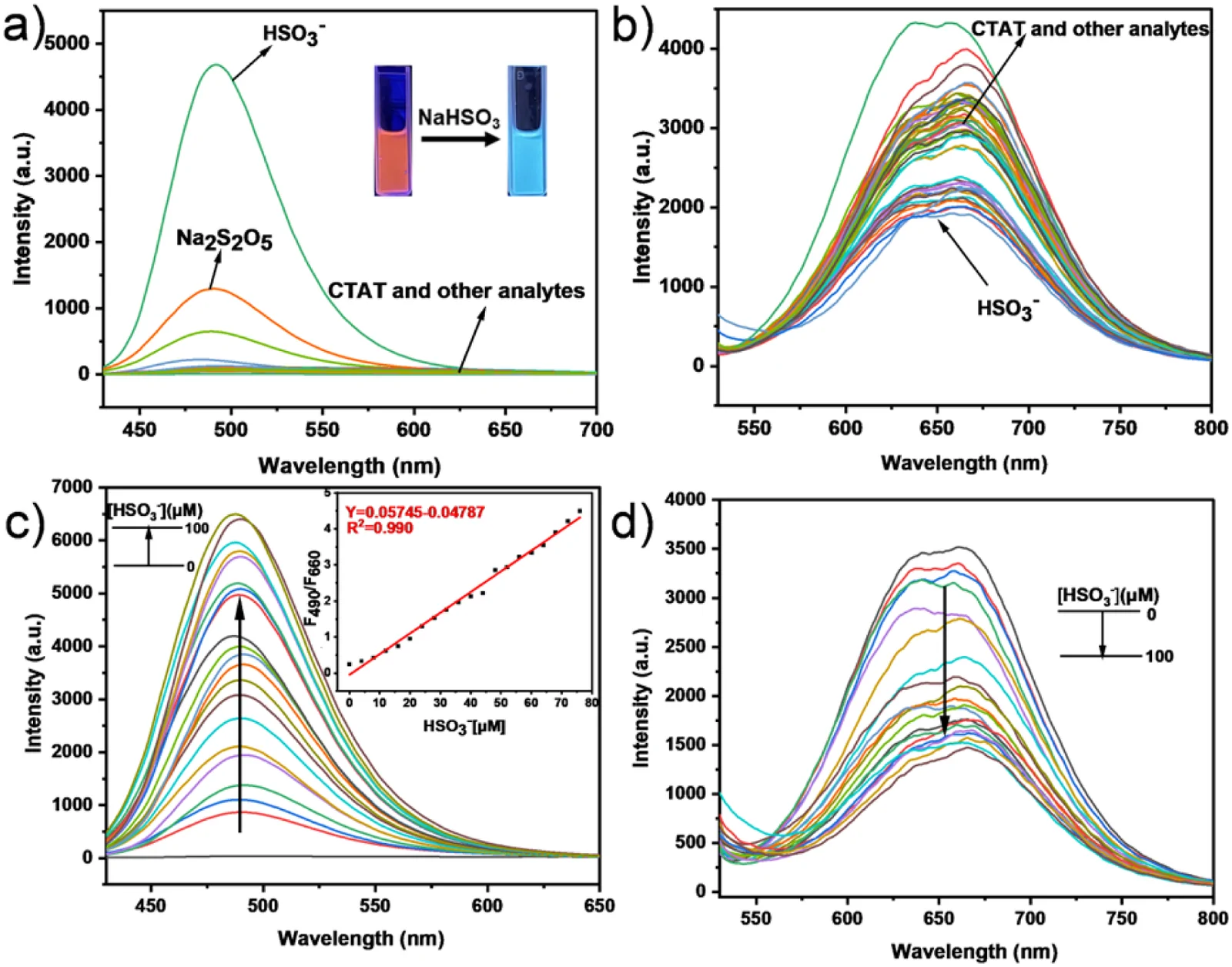
(a and b) Fluorescence spectra of probe CTAT (10 µM) with various analytes. (a): *λ*_ex_ = 410 nm, *λ*_em_ = 490 nm, (b): *λ*_ex_ = 510 nm, *λ*_ex_ = 660 nm). Inset: image of probe CTAT with and without HSO_3_^−^ under a hand-held 365 nm lamp. (c and d) Fluorescence spectra of probe CTAT (10 µM) with different concentrations of HSO_3_^−^ (0–100 µM). (c): *λ*_ex_ = 410 nm, *λ*_em_ = 490 nm, (d): *λ*_ex_ = 510 nm, *λ*_ex_ = 660 nm. Inset: the linear relationship of the fluorescence intensity ratio (*F*_490_/*F*_660_) of probe CTAT on HSO_3_^−^ concentration.

### pH and time-dependent response

The pH stability of the probe and its applicable pH range are essential factors in complex microenvironments. As such, we measured the changes in the fluorescence intensity ratio (*F*_490_/*F*_660_) of CTAT (10 µM) in the absence and presence of HSO_3_^−^ (100 µM) at different pH conditions. As illustrated in Fig. 3a, a change of pH from 2.0 to 13.0 had little impact on the fluorescence ratio (*F*_490_/*F*_660_) of CTAT, indicating that CTAT exhibits good stability. With the addition of HSO_3_^−^ (100 µM), the fluorescence intensity ratio (*F*_490_/*F*_660_) exhibited excellent enhancement over a pH range from 7.0 to 10.0, indicating that the probe CTAT can respond to HSO_3_^−^ from neutral to alkaline conditions. Coincidentally, the environment of the ER is neutral to slightly alkaline, indicating that the CTAT probe is suitable for the detection of HSO_3_^−^ in the ER under physiological conditions.

**Fig. 3:**
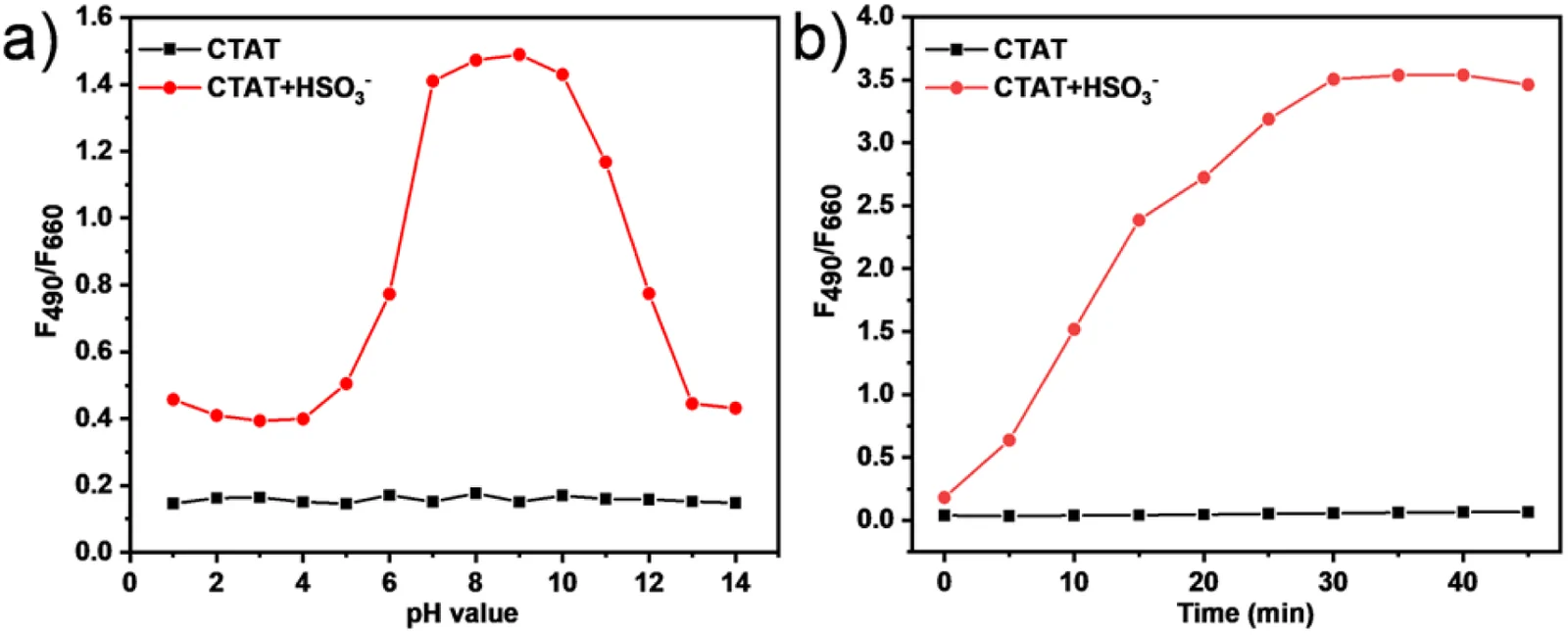
(a) Plots of *F*_490_/*F*_660_ values before and after the addition of HSO_3_^−^ to probe CTAT at different pH values (1.0–14.0). (b) Plots of *F*_490_/*F*_660_ overtime for probe CTAT with and without HSO_3_^−^.

In addition, the response time is another essential parameter in assessing the detection performance of a probe. As such we also recorded the time dependent fluorescence intensity ratio (*F*_490_/*F*_660_) of CTAT toward HSO_3_^−^. As shown in Fig. 3b, the fluorescence emission ratio (*F*_490_/*F*_660_) of CTAT exhibits minimal changes and is stable, however, after the addition of 100 µM HSO_3_^−^, the fluorescence intensity ratio (*F*_490_/*F*_660_) increased rapidly and reached a plateau within 30 min. Therefore, CTAT can serve as a stable and rapidly responsive probe for the real time monitoring of HSO_3_^−^ in the environment and living cells.

### Recognition mechanism

To verify the sensing mechanism of CTAT for detecting HSO_3_^−^, we compared the HRMS spectra of the probe before and after addition of HSO_3_^−^. As shown in Fig. 4a, a predominant peak at *m*/*z* value of 603.1732 was observed, which corresponds to [CTAT + H]^+^ (C_31_H_31_N_4_O_5_S_2_, calcd 603.1730). After the addition of HSO_3_^−^, a characteristic peak emerged at *m*/*z* 685.1462, which was attributed to [CTAT + HSO_3_^−^ + H]^+^ (C_31_H_33_N_4_O_8_S_3_, calcd = 685.1455). Combined with the spectral results, we speculate that the high nucleophilicity of HSO_3_^−^ allowed it to selectively undergo a nucleophilic addition reaction with the C=C double bond of the acrylonitrile group. As a result, the extended π-conjugation pathway between the electron donor and the electron acceptor was interrupted, which weakened the ICT effect and led the absorption and fluorescence spectra to undergo a blue-shift.

**Fig. 4:**
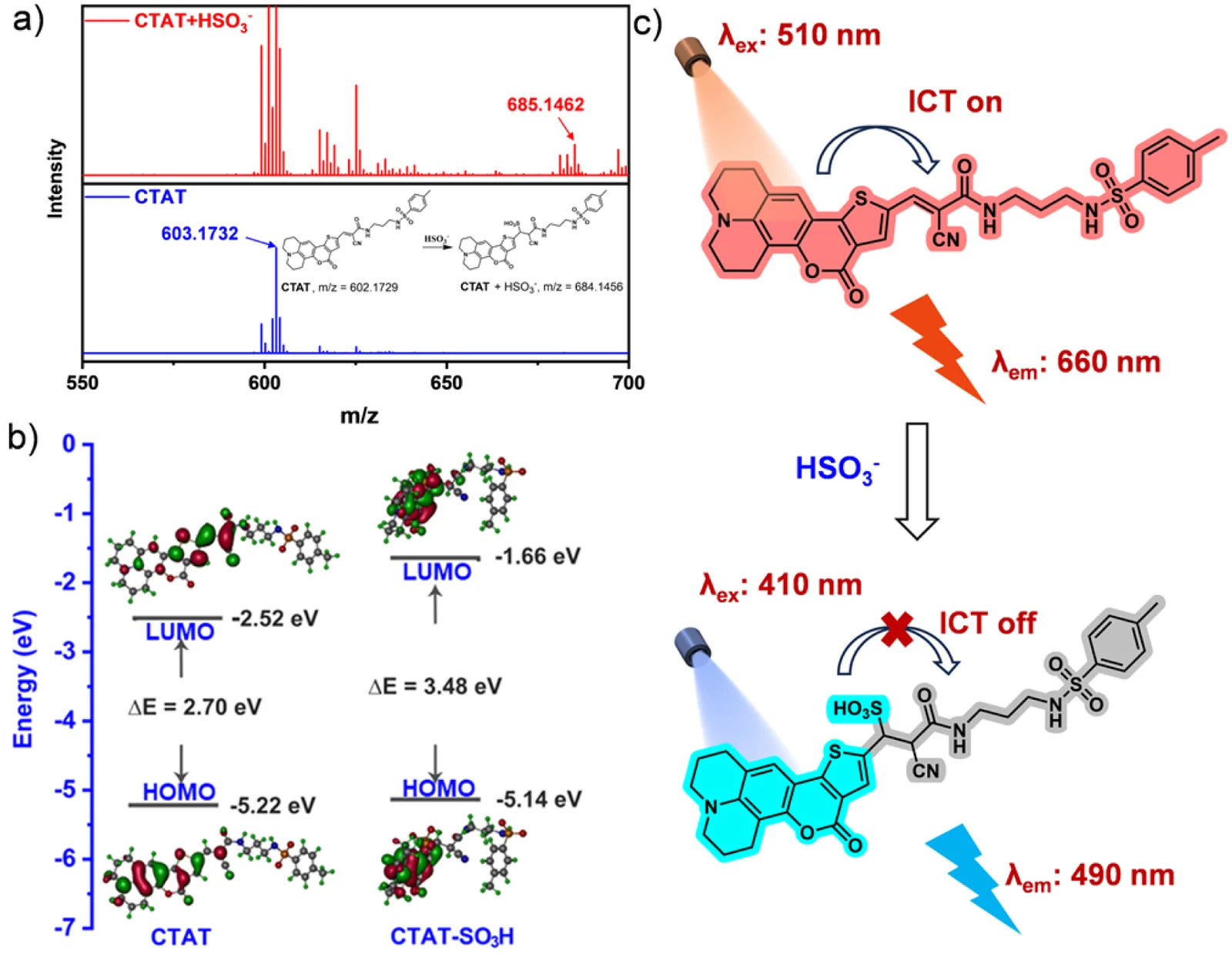
(a) ESI-MS spectra of CTAT and CTAT + HSO_3_^−^. (b) Optimized structures and HOMO/LUMO of CTAT and CTAT-SO_3_H using DFT calculations. (c) The proposed mechanism of CTAT with HSO_3_^−^.

To further investigate the sensing mechanism between HSO_3_^−^ and CTAT we used density functional theory calculations (DFT).^33^ As illustrated in Fig. 4b, The LUMO of the probe CTAT was mainly localized on the thiophene and acrylamide groups, whereas the HOMO was spread over the coumarin, reflecting an ICT process and leading the probe CTAT to emit long wavelength fluorescence. However, the HOMO and LUMO of the probe CTAT-HSO_3_^−^ were limited to the coumarin, which means that the conjugation was changed, and ICT was interrupted. Furthermore, the energy gaps between the HOMO and LUMO were calculated to be 2.70 eV and 3.48 eV for probe CTAT and probe CTAT-HSO_3_^−^, respectively. Significantly, the DFT calculations confirmed that the nucleophilic addition of HSO_3_^−^ disrupted the π conjugation of the probe CTAT and reduced the ICT, resulting in a blue shift of both the corresponding absorption and emission spectra.

### Cytotoxicity and imaging of HSO_3_^−^ and polarity in living cells

To explore the applicability of the probe for the imaging of living cells, we used HepG2 and L-02 cell cultures to assess the toxicity of probe CTAT. We were pleased to observe that within the concentration range of our study (0–50 µM), CTAT is not-obviously cytotoxic (Fig. S7). Thereafter, we investigated the ER-targeting capacity of probe CTAT*via* colocalization experiments. As illustrated in Fig. S8, the CTAT's red channel exhibited substantial overlap with the fluorescence of commercial ER-Tracker Green in both HepG2 and L-02 cells, with high Pearson correlation coefficients (0.92 and 0.90, respectively). These results demonstrate that CTAT possesses a consistent and effective ER-targeting capability in both cell lines. Moreover, the low Pearson correlation coefficients observed between CTAT and the commercial mitochondrial, Golgi, and lysosomal trackers (Fig. S8) indicate minimal nonspecific accumulation in these organelles, further supporting the ER-targeting specificity of CTAT.

Given the outstanding optical properties and good cellular safety of probe CTAT, we next evaluated the fluorescence detection capability of CTAT for HSO_3_^−^ in HepG2 cells. As shown in Fig. 5, cells were incubated with CTAT (10 µM) for 30 min and only red fluorescence was observed. Subsequently, the cells were pretreated with NaHSO_3_ (100 µM) for 30 min and then incubated with CTAT (10 µM) for 30 min. Consistent with our spectroscopic experiments, a remarkable enhancement of fluorescence in the blue channel was observed and the fluorescence in the red channel disappeared. We further studied the imaging ability of CTAT for endogenous HSO_3_^−^ by incubation of HepG2 cells with GSH and Na_2_S_2_O_3_. The cells were treated with GSH (500 µM) for 30 min and then probe CTAT was added for another 30 min, and the fluorescence intensities in the two channels remained almost unchanged. However, when the cells were incubated with GSH (500 µM) and Na_2_S_2_O_3_ (an inducer to produce endogenous HSO_3_^−^, 250 µM)^2,34^ for 30 min respectively, and then probe CTAT was added for 30 min, the fluorescence signal in the blue channel was significantly enhanced and the red fluorescence was significantly quenched (Fig. S9). These results indicate that CTAT can image exogenous and endogenous HSO_3_^−^ in living cells.

**Fig. 5:**
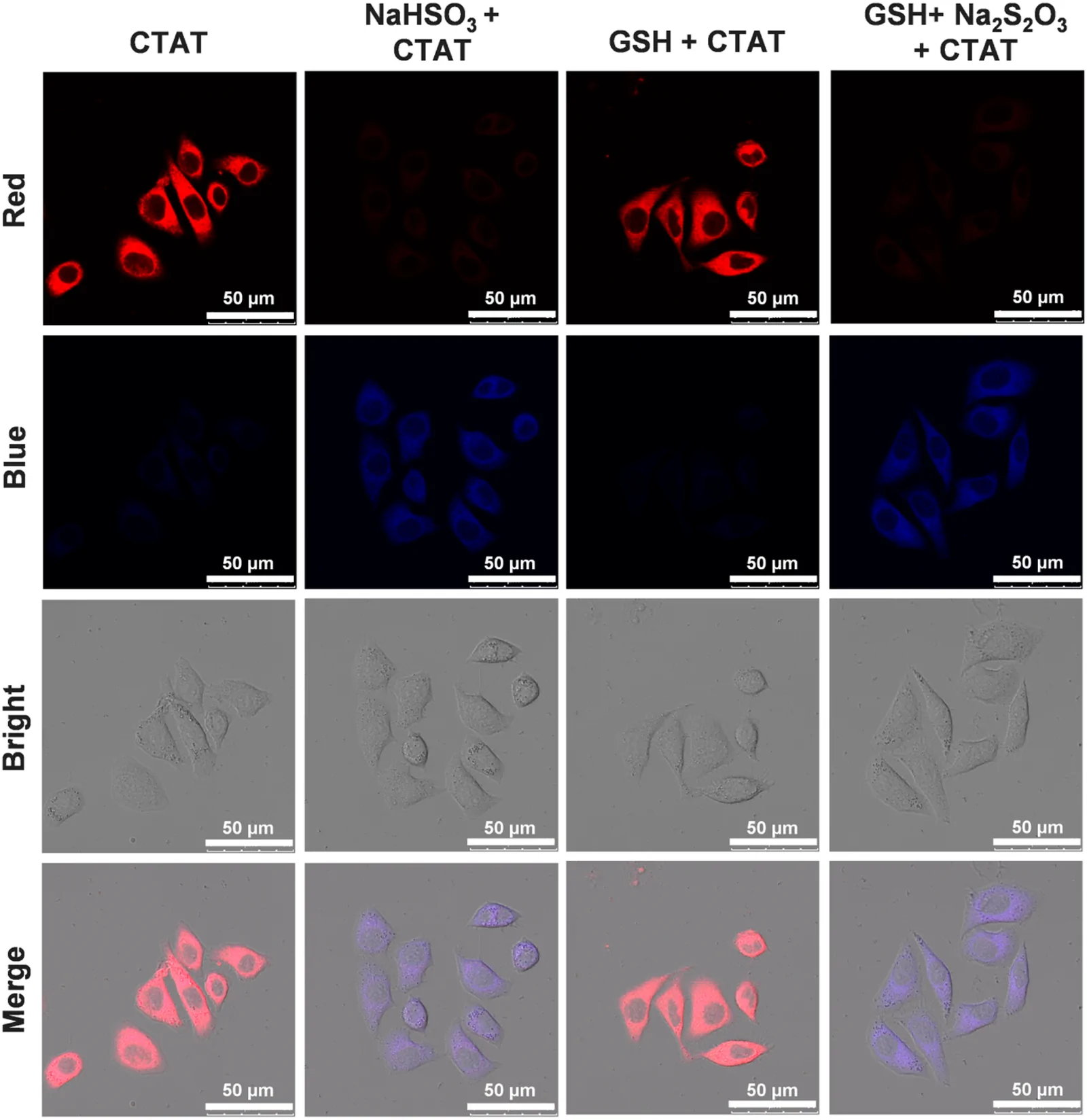
Confocal imaging of the HSO_3_^−^ levels with CTAT inside cultured HepG2 cells. First column (left to right): incubation with 10 µM CTAT, second column: incubation with 10 µM CTAT and 100 µM HSO_3_^−^, third column: incubation with 10 µM CTAT and 500 µM GSH, fourth column: incubation with 10 µM CTAT, 500 µM GSH and 250 µM Na_2_S_2_O_3_. Top row red channel (*λ*_ex_/*λ*_em_ = 514/600–680 nm), second row blue channel (*λ*_ex_/*λ*_em_ = 405/480–530 nm), third row bright field, bottom row merged. Scale bar is 50 µm.

To assess the ability of CTAT to monitor polarity changes, HepG2 cells were cultured with different concentrations of glutamate (Glu) (50, and 100 µM), which can increase cellular polarity,^35,36^ and afterward incubated with CTAT. The red channel fluorescence decreased significantly as the concentration of the Glu increased, indicating the capacity of CTAT to detect variations in the polarity of living cells (Fig. 6 and S10a). Additionally, extensive research has shown that the microenvironment of cancer cells significantly differs from that of normal cells. Cancer cells exhibit lower polarity compared to normal cells. Thus, we evaluated the fluorescence imaging capacity of CTAT (10 µM) in cancer cells (HepG2) and normal cells (L-02). As shown in Fig. S10b, HepG2 cells exhibited significantly higher fluorescence intensity than L-02 cells, and the mean fluorescence intensity in cancer cells was approximately 1.5 times that of normal cells. These observations validate the capability of CTAT to detect variations in HSO_3_^−^ and polarity inside the ER of living cells.

**Fig. 6:**
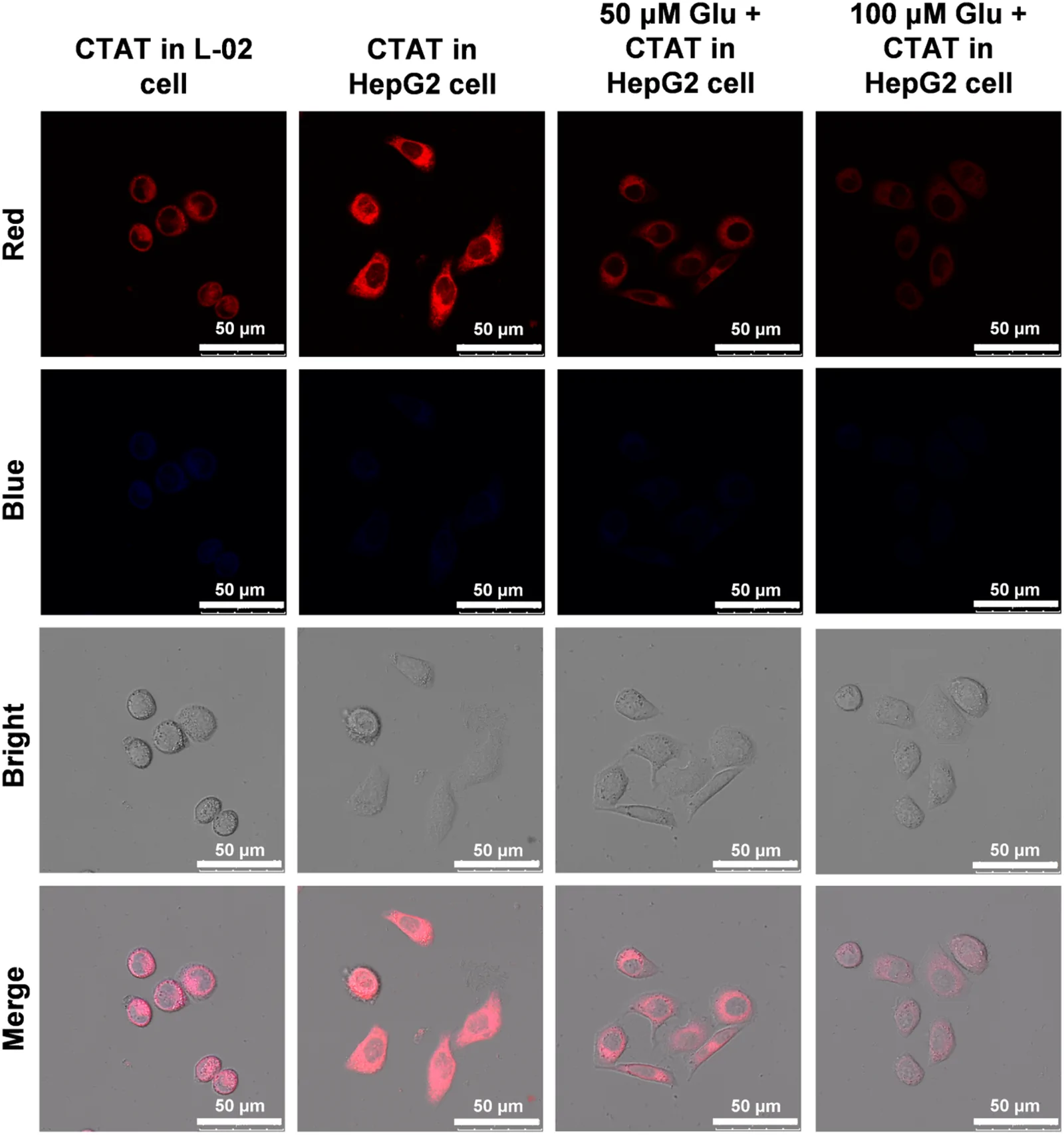
Confocal imaging of CTAT for monitoring intracellular polarity. First column (left to right): incubation with 10 µM CTAT in L-02 cell, second column: incubation with 10 µM CTAT in HepG2 cell, third column: incubation with 10 µM CTAT and 50 µM Glu in HepG2 cell, fourth column: incubation with 10 µM CTAT and 100 µM Glu in HepG2 cell. Top row red channel (*λ*_ex_/*λ*_em_ = 514/600–680 nm), second row blue channel (*λ*_ex_/*λ*_em_ = 405/480–530 nm), third row bright field, bottom row merged. Scale bar is 50 µm. (b) Mean fluorescence intensity of the red channel in L-02 and HepG2 cells. (c) Mean fluorescence intensity of the red channel in different groups of HepG2 cells.

### Imaging of zebrafish

In view of the above-described outstanding fluorescence performance of CTAT, we further exploited the capability of CTAT for the fluorescence imaging of HSO_3_^−^ and polarity in zebrafish. First, we evaluated the ability of CTAT to detect HSO_3_^−^ in zebrafish. Fig. 7 shows that when the zebrafish were incubated with only CTAT (10 µM) red fluorescence was observed. However, when the zebrafish were pretreated with NaHSO_3_ and CTAT, the fluorescence intensity in the blue channel was significantly enhanced, while the fluorescence signal in the red channel was reduced (Fig. S11). The same experimental phenomenon was also observed when zebrafish were incubated with GSH and Na_2_S_2_O_3_ (an inducer to produce endogenous HSO_3_^−^), and then probe CTAT. However, when zebrafish were incubated with GSH and CTAT, only red fluorescence signals were observed. These results indicate that CTAT has the ability to image exogenous and endogenous HSO_3_^−^ in organisms.

**Fig. 7:**
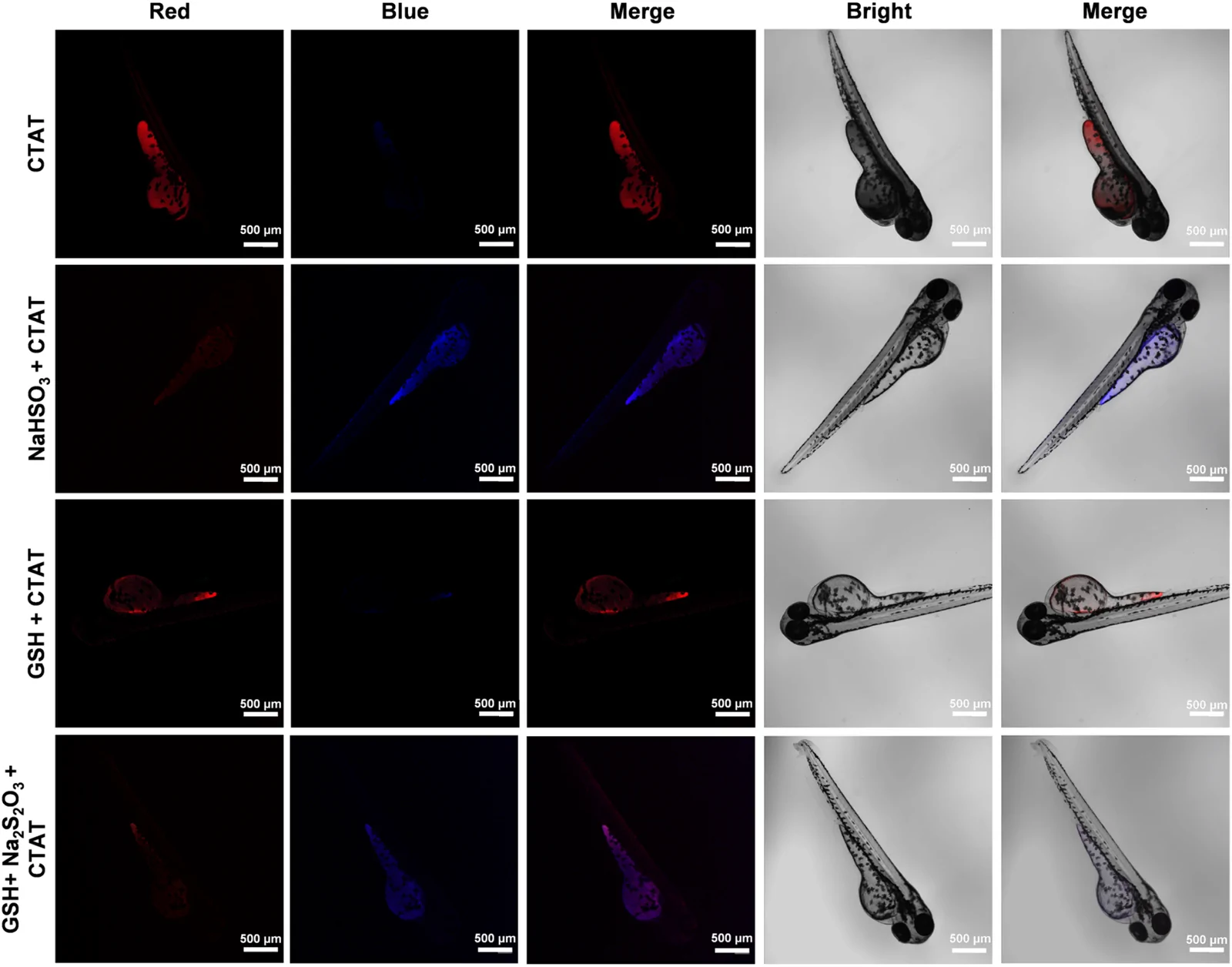
Confocal imaging of the HSO_3_^−^ levels with CTAT in zebrafish. First column (left to right): red channel (*λ*_ex_/*λ*_em_ = 514/600–680 nm), second column: blue channel (*λ*_ex_/*λ*_em_ = 405/480–530 nm), third column: merged, fourth column: bright field, fifth column: merged. Top row: incubation with 10 µM CTAT, second row: incubation with 10 µM CTAT and 100 µM HSO_3_^−^, third row: incubation with 10 µM CTAT and 500 µM GSH, bottom row: incubation with 10 µM CTAT, 500 µM GSH and 250 µM Na_2_S_2_O_3_. Scale bar is 500 µm.

Moreover, we further explored the ability of the probe CTAT to visualize polarity changes in zebrafish during different growth periods.^36,37^ As shown in Fig. 8, the embryos of zebrafish incubated with CTAT exhibited strong fluorescence in the red channels, whereas adult zebrafish exhibited weaker fluorescence (Fig. S12a). Additionally, with an increase in Glu concentration, the fluorescence intensity of adult zebrafish gradually decreases (Fig. S12b). These results indicate that CTAT can detect changes in the polarity of zebrafish during growth.

**Fig. 8:**
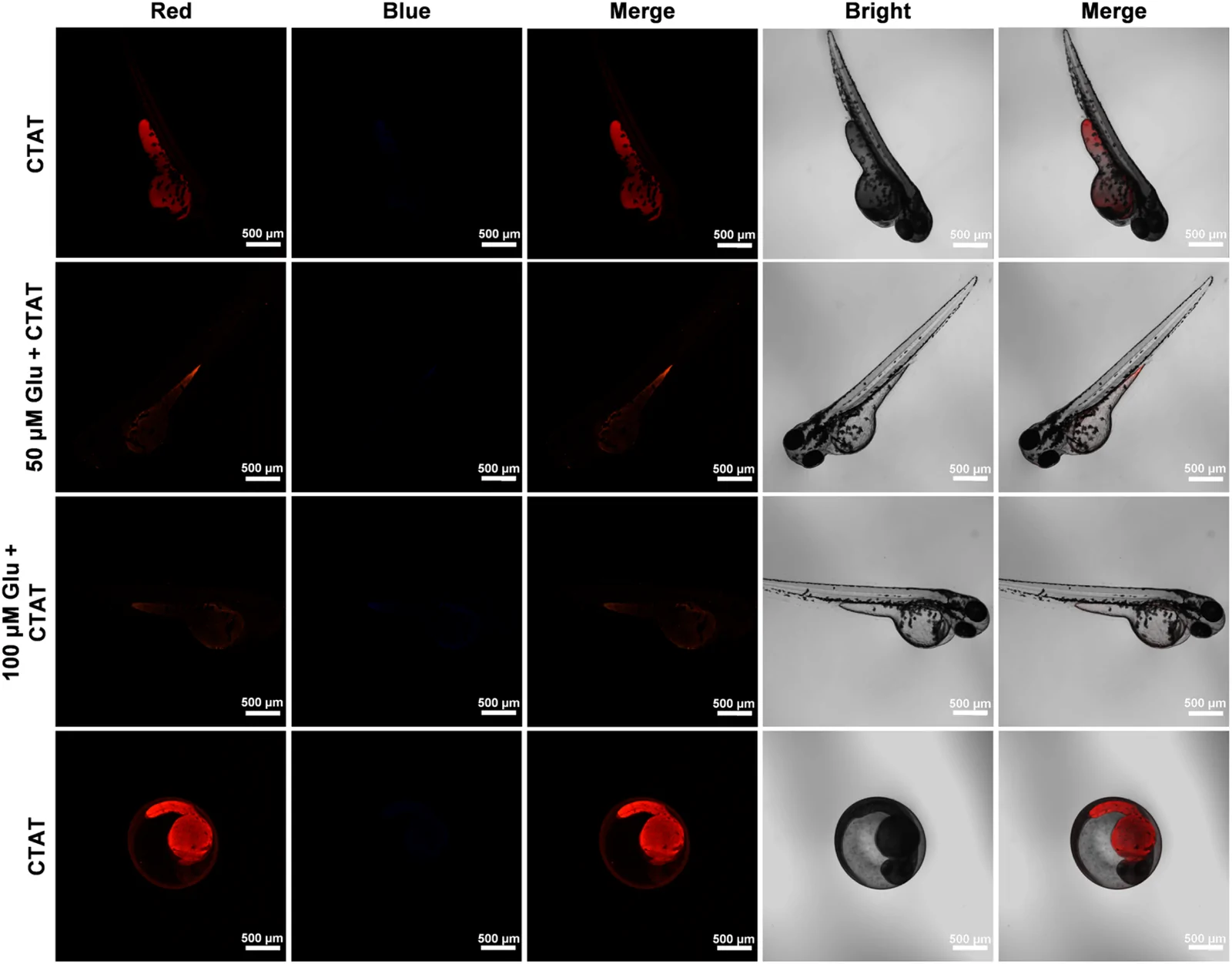
Confocal imaging of CTAT for monitoring intracellular polarity. First column (left to right): red channel (*λ*_ex_/*λ*_em_ = 514/600–680 nm), second column: blue channel (*λ*_ex_/*λ*_em_ = 405/480–530 nm), third column: merged, fourth column: bright field, fifth column: merged. Top row: incubation with 10 µM CTAT in adult fish, second row: incubation with 10 µM CTAT and 50 µM Glu in adult fish, third row: incubation with 10 µM CTAT and 100 µM Glu in adult fish, bottom row: incubation with 10 µM CTAT in fish embryo. Scale bar is 500 µm.

### Imaging of mice

To further illustrate that CTAT can distinguish polarity levels *in vivo*, we constructed a tumor model and an LPS-induced mouse peritoneal inflammation model (All animal procedures were performed in accordance with the Guidelines for Care and Use of Laboratory Animals of Xi'an Medical University and approved by the Animal Ethics Committee of Xi'an Medical University, No: XYLS2026196). As shown in Fig. S13, CTAT was injected into tumor tissue and normal tissue respectively, and then incubated for 10 min for imaging. The results indicated that the fluorescence signal in the tumor tissues was significantly greater than that in normal tissues, which is consistent with above cell experiments. Moreover, we further evaluated the imaging ability toward polarity in an LPS-induced inflammatory model. The mice were sequentially injected intraperitoneally with LPS (100 µL, 1 mg mL^−1^, 12 h) and CTAT (100 µL, 50 µM, 30 min), followed by fluorescence imaging. As shown in Fig. 9, stronger fluorescence was observed in mice that received LPS pretreatment prior to CTAT treatment, relative to the CTAT-only group. The above results indicate that CTAT can sensitively visualize polarity-associated changes in tumor and inflammatory tissues *in vivo*. Considering the potential relationship between polarity alterations and changes in the ER microenvironment, CTAT may serve as a promising imaging probe for reporting disease-associated alterations in the ER microenvironment, with potential utility in the detection and monitoring of pathological conditions related to ER dysfunction.

**Fig. 9:**
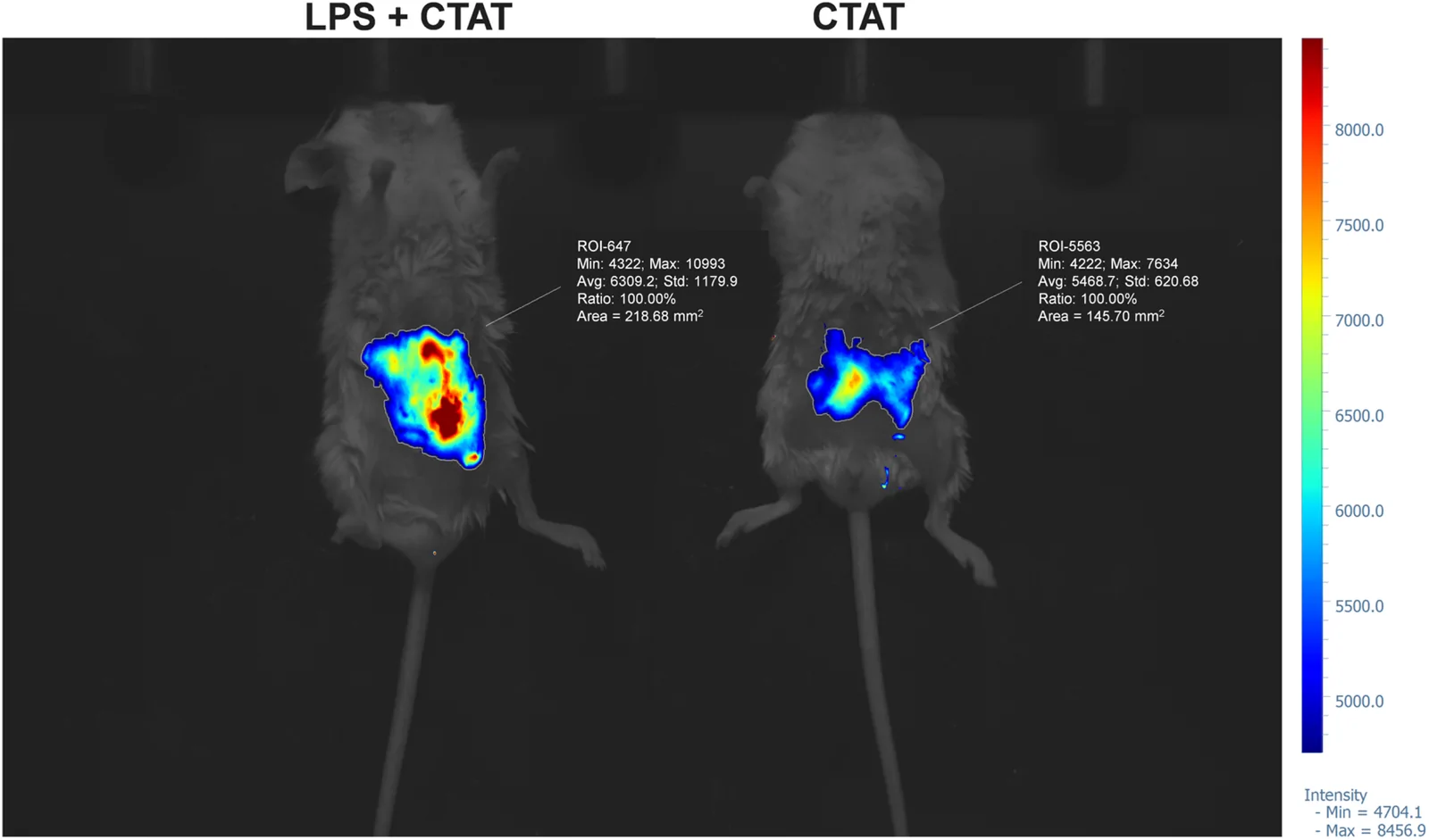
*In vivo* fluorescence imaging of the polarity in LPS-induced mouse peritoneal inflammation model. Left is model, right is control.

Finally, we focused our attention on whether CTAT could be used to detect HSO_3_^−^ in a drug-induced liver injury (DILI) model. Acetaminophen (APAP) is an antipyretic and analgesic drug; excessive intake can trigger hepatotoxicity, induce oxidative stress, and generate large amounts of biothiols and HSO_3_^−^ in the liver.^38^ As shown in Fig. S14a, compared with the control group, the liver injury group exhibited higher levels of both alanine aminotransferase (ALT) and aspartate aminotransferase (AST). Consistently, the liver injury group also exhibited significantly enhanced blue fluorescence and markedly weakened red fluorescence compared with the control group (Fig. 10). NAC possesses antioxidant activity and can alleviate oxidative stress, thereby attenuating APAP-induced liver injury. Accordingly, compared with the injury group, the NAC group exhibited decreased blue fluorescence intensity and increased red fluorescence intensity (Fig. S14b and c). Collectively, these results demonstrate that CTAT can sensitively report APAP-induced biochemical alterations in liver tissue. Given the close association between oxidative injury, HSO_3_^−^ dysregulation, and ER stress, CTAT may serve as an early-warning and diagnostic imaging tool for detecting and monitoring ER stress-related hepatic injury.

**Fig. 10:**
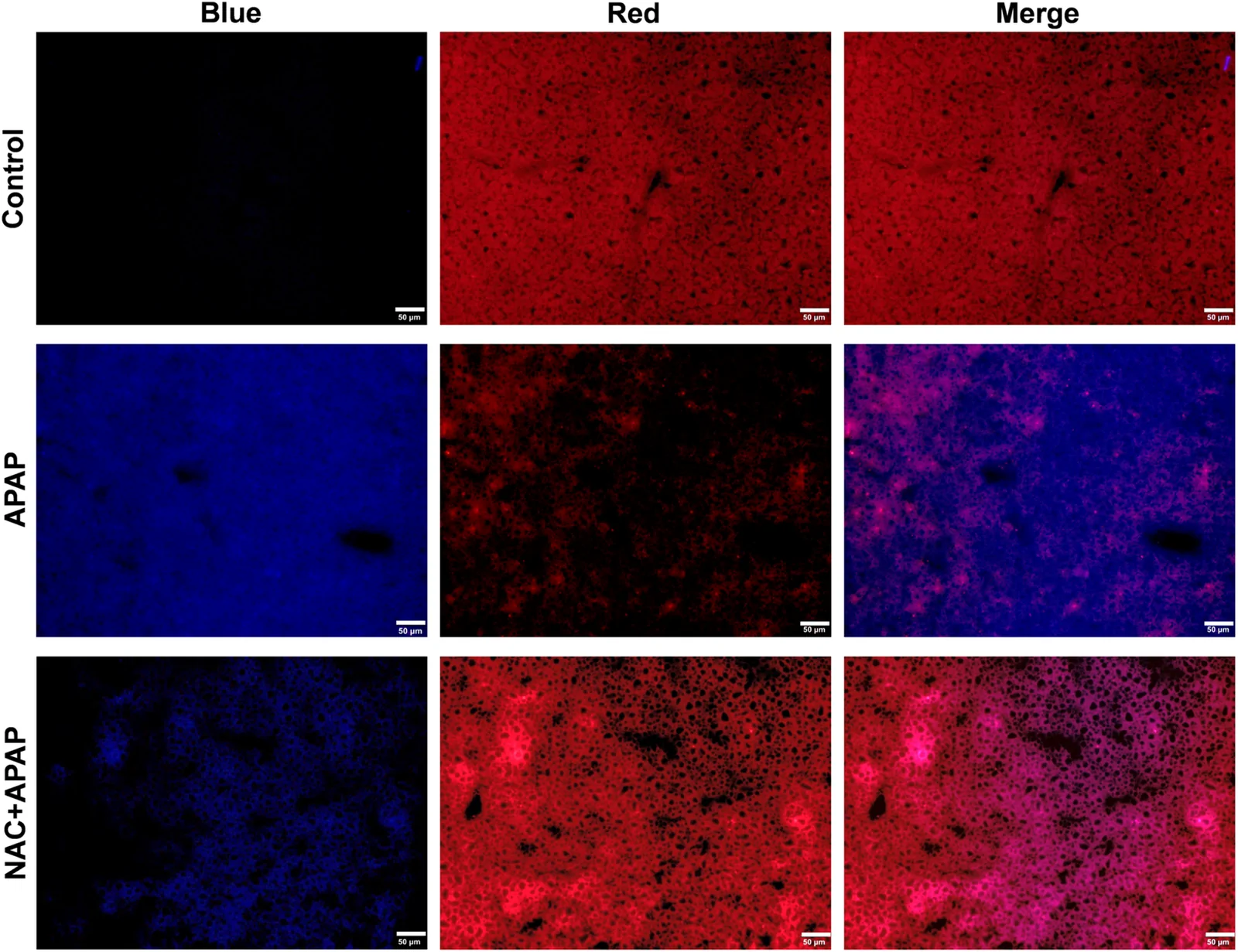
Fluorescence images of liver slices. (Top row: control group, middle row: APAP group, bottom row: APAP + NAC group. First column: red channel: *λ*_ex_/*λ*_em_ = 405/480–530 nm, second column: blue channel: *λ*_ex_/*λ*_em_ = 514/600–680 nm, third column: merged). Scale bar: 50 µm.

## Conclusions

In summary, we have developed a promising dual-excitation ratiometric ER targeted fluorescent probe CTAT for HSO_3_^−^ and polarity detection. Gratifyingly, CTAT enabled the crosstalk-free sensing of HSO_3_^−^ and polarity, owing to the distinct spectral responses: detecting HSO_3_^−^ through an effective ratiometric mechanism (emission at 490 nm) while responding to polarity *via* a weakened red fluorescence signal (emission at 660 nm). The large differences in wavelengths (150 nm) between the two biomarkers ensures minimal spectral interference. Furthermore, probe CTAT was successfully employed to achieve the concurrent monitoring of endogenous/exogenous HSO_3_^−^ fluctuations and polarity variations in live cells and zebrafish. Most strikingly, CTAT exhibited a highly-sensitive and selective response to fluctuations in polarity across an extensive array of *in vivo* disease models, including APAP-induced liver injury, LPS-induced peritoneal inflammation and cancer. Collectively, we anticipate that CTAT will become a powerful and potential multi-functional detection tool for the clinical diagnosis and treatment monitoring of various diseases.

## Experimental

The detailed information of materials, instruments, synthesis and characterization of CTAT, cell culture, and *in vitro*/*in vivo* experiments can be found in the SI.

## Author contributions

Hao Wu: validation, writing – original draft & editing. Yue Sun: data curation, writing – original draft. Yuan Wang: investigation, supervision, project administration. Jing Zhang: conceptualization, funding acquisition. Wei-Na Wu: formal analysis, methodology. Zhi-Hong Xu: investigation, supervision, project administration. Tony D. James: visualization, writing – review & editing.

## Conflicts of interest

No potential conflict of interest was reported by the authors.

## Supplementary Material

SC-OLF-D6SC05911B-s001

## Data Availability

All relevant data obtained during this research are available within the manuscript and supplementary information (SI). Supplementary information is available. See DOI: https://doi.org/10.1039/d6sc05911b.
