# Supplementary material for: A bifunctional ER-targeting fluorescent probe for the detection of SO2 and polarity in living cell and animals
Source: Chem Sci. 2026 Oct 14. Online ahead of print. doi: 10.1039/d6sc05911b (PMC13662754; doi:10.1039/d6sc05911b)
Supplement: Supplementary file 1 [file SC-OLF-D6SC05911B-s001.pdf]

Supplementary Information (SI) for Chemical Science.
This journal is © The Royal Society of Chemistry 2026
Supplementary Information (SI) for Chemical Science.
This journal is © The Royal Society of Chemistry

A bifunction ER-targetable fluorescent probe for the detection of SO_2 and polarity in living cell and animals

Hao Wu[a,†], Yue Sun[a,†], Yuan Wang[b,*], Jing Zhang[a,*], Wei-Na Wu[b], Zhi-Hong Xu[c,d*], Tony D. James[e,f,*]

[a] *Translational Research on Cell and Gene Therapy for Higher Education Institutions for Shaanxi key Laboratory of Higher Education Institutions of Shaanxi, Xi'an Medical University, Xi'an, Shaanxi 710021, PR China*

[b] *College of Chemistry and Chemical Engineering, Henan Key Laboratory of Coal Green Conversion, Henan Polytechnic University, Jiaozuo 454000, PR China*

[c] *Key Laboratory of Chemo/Biosensing and Detection, College of Chemical and Materials Engineering, Xuchang University, Xuchang, 461000, PR China*

[d] *College of Chemistry, Zhengzhou University, Zhengzhou, 450052, PR China*

[e] *Department of Chemistry, University of Bath, Bath, BA2 7AY, UK*

[f] *School of Chemistry and Chemical Engineering, Henan Normal University, Xinxiang. 453007, PR China*

*Corresponding author. Tel.: +86 391 3987818; Fax: +86 391 3987811; e-mail: wangyuan08@hpu.edu.cn (Y. Wang); jingzhang@xiyi.edu.cn (J. Zhang); xuzhihong1980@xcu.edu.cn (Z.-H. Xu); t.d.james@bath.ac.uk (T. D. James).

†These authors contributed equally to this work.

1 Experimental section

1.1. Materials and instrumentation

Solvents and starting materials for the synthetic procedures were purchased commercially and used as received. ER-Tracker Green, LPS, GSH, Glutamate (Glu) and cell counting kit-8 was commercially available from Yeasen Biotechnology (Shanghai) Co., Ltd. Dulbecco's Modified Eagle Medium (DMEM basic 1×) Medium, phosphate buffered saline (PBS), penicillin and streptomycin were purchased from Servicebio Company (Wuhan, China). ^{1}H NMR and ^{13}C NMR spectra were measured by Bruker Avance 400 spectrometer in DMSO-d_6 solution. High-resolution electrospray ionization mass spectra (HR-ESI-MS) were measured by Thermo Scientific Q Exactive instrument (Bremen, Germany). The UV spectra were recorded on a METASH Q6 spectrophotometer (Shanghai). Fluorescence spectra were determined on an F97 fluorescence spectrophotometer made by Shanghai Lengguang Technology Co. Ltd.

1.2. Synthesis and Characterization

Compound **A** (4-oxo-7,8,11,12-tetrahydro-4H,6H,10H-pyrido[3,2,1-ij]thieno[2',3':4,5]pyrano[2,3-f]quinoline-2-carbaldehyde, 2 mmol),[1] compound **B** (2-cyano-N-(3-((4-methylphenyl)sulfonamido)propyl)acetamide, 3 mmol),[2-3] were dissolved in 10 mL of EtOH. The mixture was heated and refluxed at 80 °C for 4 h. After the reaction was completed, it was cooled to room temperature, filtered, and recrystallized to obtain red powder solid **CTAT** (0.94 g, Yield: 78%). ^{1}H NMR (400 MHz, DMSO-d_6) δ (ppm): 8.34 (s, 1H), 8.28 (t, J = 5.2 Hz, 1H), 8.12 (s, 1H), 7.68 (d, J = 8.1 Hz, 2H), 7.54 (t, J = 5.6 Hz, 1H), 7.39 (d, J = 8.0 Hz, 2H), 7.15 (s, 1H), 3.30 – 3.23 (m, 4H), 3.19 (d, J = 5.8 Hz, 2H), 2.74 (s, 6H), 2.37 (s, 3H), 1.88 (s, 4H), 1.68 – 1.57 (m, 2H). ^{13}C NMR (100 MHz, DMSO-d_6) δ (ppm) 161.04, 154.44, 149.53, 146.82, 143.51, 143.05, 138.04, 137.96, 132.13, 130.11, 127.00,

121.80, 119.94, 119.79, 117.06, 106.39, 103.87, 101.41, 49.84, 49.26, 37.86, 29.48, 27.08, 21.41, 21.10, 20.56, 20.28. HRMS (ESI, m/z) for $[C_{31}H_{31}N_4O_5S_2]^+$: calcd 603.1736, found 603.1732.

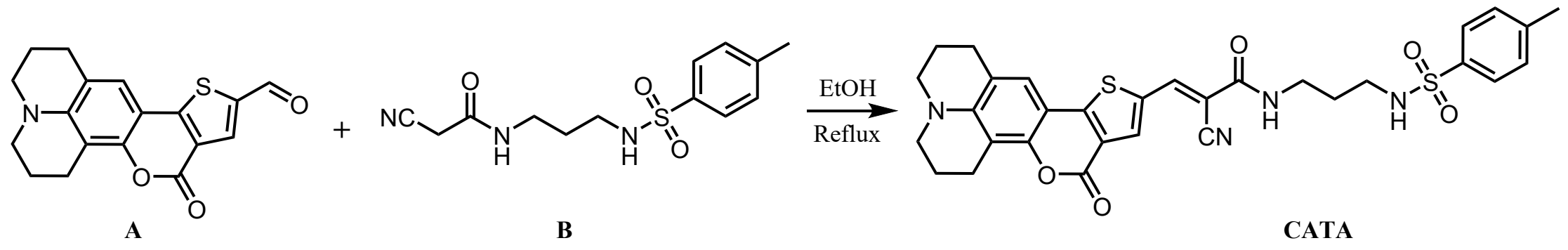

Scheme S1. Synthetic route for **CTAT**

1.3. Fluorescence spectral measurements

The spectral analyses were performed in PBS buffer at room temperature. The concentration of the probe **CTAT** for the fluorescence measurements was 10 μM. Stock solutions of HSO_3^- and other common analytes were prepared in distilled water. The stock solutions were used immediately and then diluted to the desired concentrations as needed. Fluorescence spectrophotometric titrations were conducted directly in a 2 mL cuvette by successive addition of the corresponding chemical reagent using a microliter syringe. Upon addition of every aliquot, the solution was well mixed then the spectrum was recorded.

1.4. Cell culture and Animal model

HepG2 and L-02 cells were originally obtained from the American Type Culture Collection (ATCC). The cells were cultured in DMEM medium with 10% fetal bovine serum at 37 °C in a humidified atmosphere containing 5% CO_2. Zebrafish were obtained from Shanghai FishBio Co., Ltd., (China). Female Balb/c mice (6-8 weeks) weighing approx. 20 g were supplied by the Hunan Silek Jingda Experimental Animal Co., Ltd. All *in vivo* experiments were performed in compliance with the Institutional Animal Care and Use Committee of Xi'an Medical University (XYLS2026196).

1.5. In Vitro Cell Cytotoxicity Assay

HepG2 cells and L02 were seeded into a 96-well plate (0.5 × 10^4 cells per well), and then incubated in 100 μL DMEM medium containing 10% FBS for 12 h. The cells were treated with different **CTAT** concentrations, and the cell cytotoxicity were evaluated by CCK8 assay.

1.6. Cell Colocalization

In order to further prove the mitochondrial targeting effect of the **CTAT**, **CTAT** (110 μM) was added to HepG2 cells for 30 min. The treated cells were pre-stained with 10 μM ER-tracker green, and imagined using a confocal laser scanning microscope (CLSM) (TCSSP8, Leica, Germany).

1.7. Fluorescence Imaging of HSO_3^- Sensing by CTAT

To explore the ability of the **CTAT** for sensing HSO_3^- in cells, the HepG2 cells were treated with HSO_3^- (100 μM) for 30 min, washed with PBS buffer, then 10 μM **CTAT** was incubated with the cells for another 30 min. Moreover, $Na_2S_2O_3$ and GSH were used to stimulate cells to produce endogenous, which can be verified the ability of endogenous SO_2 detection of **CTAT**. In brief, 500 μM GSH and 250 μM $Na_2S_2O_3$ for was co-incubated for 30 min before the treatment of 10 μM **CTAT**. The HepG2 cells were imagined by CLSM.

Furthermore, we have verified the sensing HSO_3^- ability of **CTAT** in APAP-induced acute liver injury model. Experiments using male BALB/c mice (5-6 weeks old, 20-22 g), which were randomly divided into 3 groups (6 per group), including a normal group, an APAP (300 mg kg^{-1}) treatment group, and an APAP+NAC group (NAC pre-treated) All animals fasted for12 h but had free access to water before administering APAP. APAP group: Mice were intraperitoneally injected with APAP (200 μL, 300 mg/kg). NAC group: 2 h before APAP injection, NAC (200 μL, 100 mg/kg) were intraperitoneally injected. And the control group mice were intraperitoneally injected with saline. After 24 hours, mice

were anesthetized with isofluorane, and blood and tissue samples were analyzed. The levels of alanine aminotransferase (ALT) and aspartate aminotransferase (AST) in the serum of mice were measured in the normal group and the APAP group. Subsequently, the prepared frozen sections were incubated with **CTAT** (20 μM) at 37 °C for 30 min, washed with PBS three times. Then collected fluorescence microscopy images (IX73, OLYMPUS, Japan).

1.8. Imaging of polarity in Cells

To explore the ability of the **CTAT** for sensing polarity in cells, **CTAT** (10 μM) was co-incubated with L-02 and HepG2 cells for 30 minutes respectively, and then imaged after three times washed with PBS buffer. Moreover, intracellular polarity increases with glutamate (Glu) concentration, thus validating the ability of **CTAT** to endogenously detect changes in polarity. Briefly, the HepG2 cells were treated with Glu (50μM and 100 μM, respectively) for 30 min, washed with PBS buffer, then 10 μM **CTAT** was incubated with the cells for another 30 min and washed with PBS buffer. Finally, cells were imagined by CLSM.

1.9. Polarity Detection of CTAT probe in Tumor Model and LPS-induced mouse peritoneal inflammation model

4T1-bearing mice model was established by the injection of 1×10^6 4T1 cells into mice subcutaneously. When the tumor reached ~100 mm^3, 20 μM **CTAT** was injected intratumorally into the tumor site and the corresponding normal area. Fluorescence signals were detected and analyzed using the IVIS system

Furthermore, LPS-induced abdominal inflammation model will change the polarity of abdominal epithelial cells. Generally, the mice are sequentially injected intraperitoneally with LPS (100 μL, 1mg/mL, 12 h) and **CTAT** (100 μL, 50 μM, 30 min), followed by fluorescence imaging (LumiFluor AVIS LDX 800, Koreshine Faye, Beijing).

1.10. Imaging of polarity in Cells

For zebrafish, all the steps were conducted in agreement with the above procedures.

2. Figures:

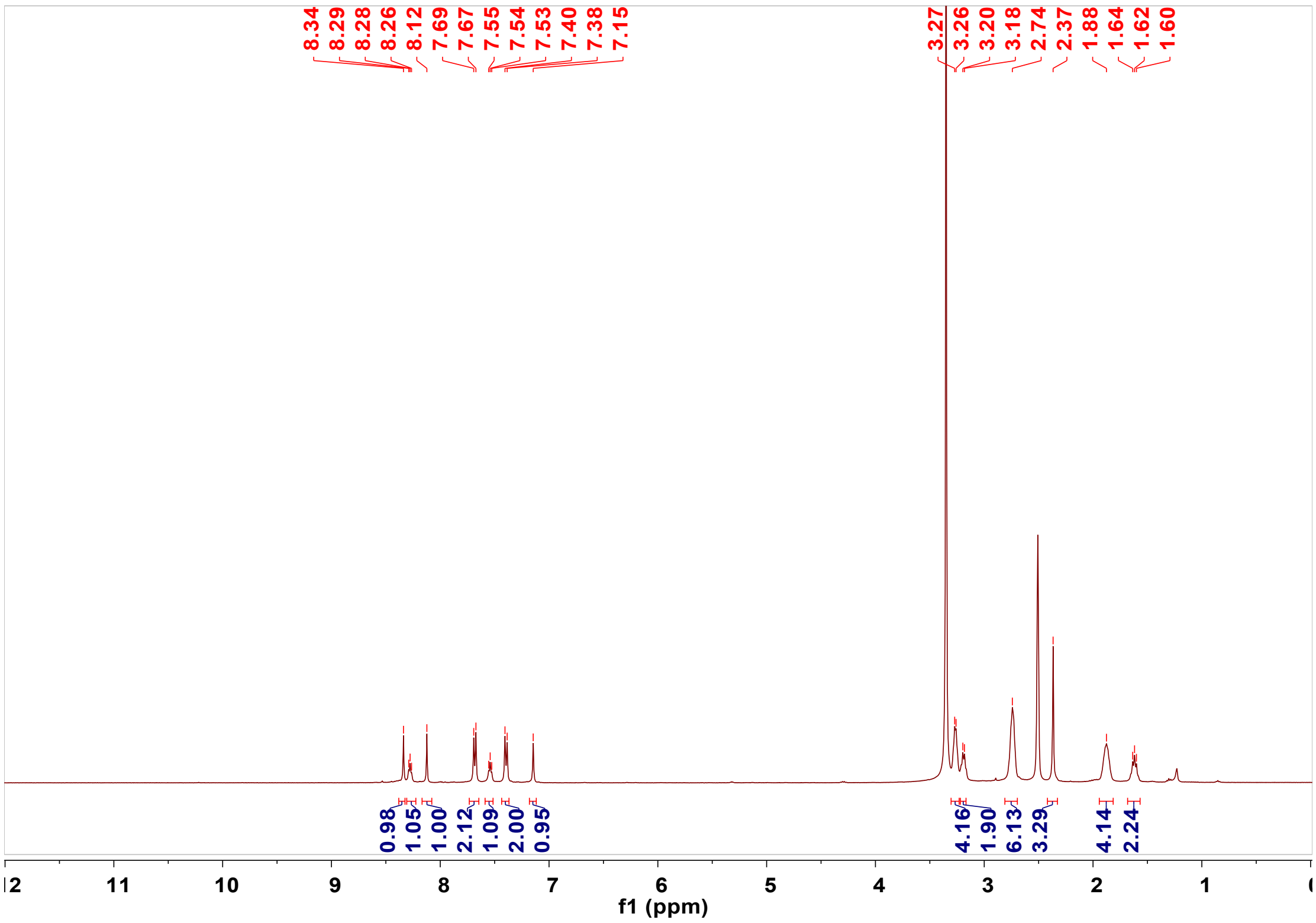

Fig. S1. ^{1}H NMR spectrum of **CTAT** in DMSO-d_6 solution.

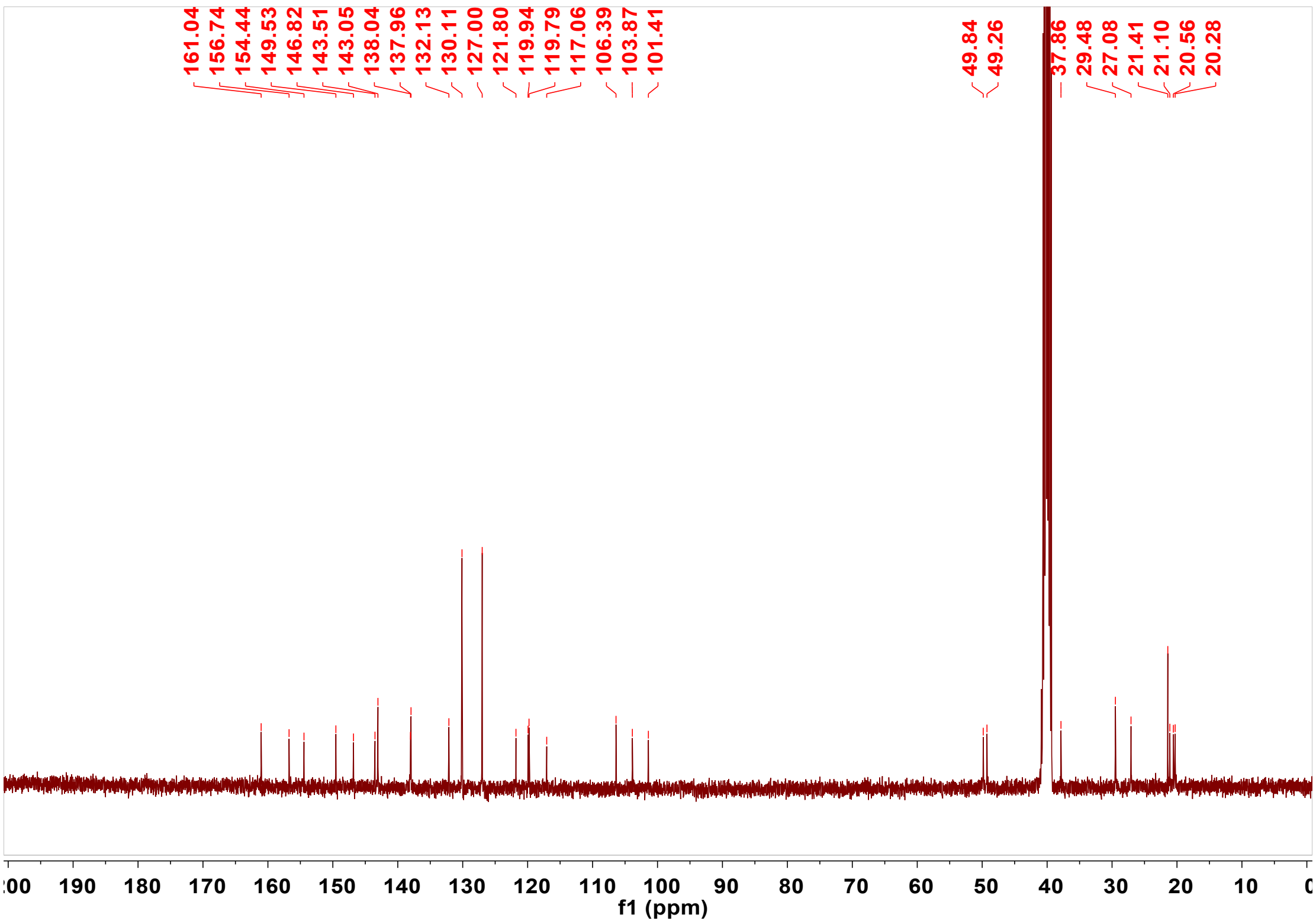

Fig. S2. ^{13}C NMR spectrum of **CTAT** in DMSO-d_6 solution.

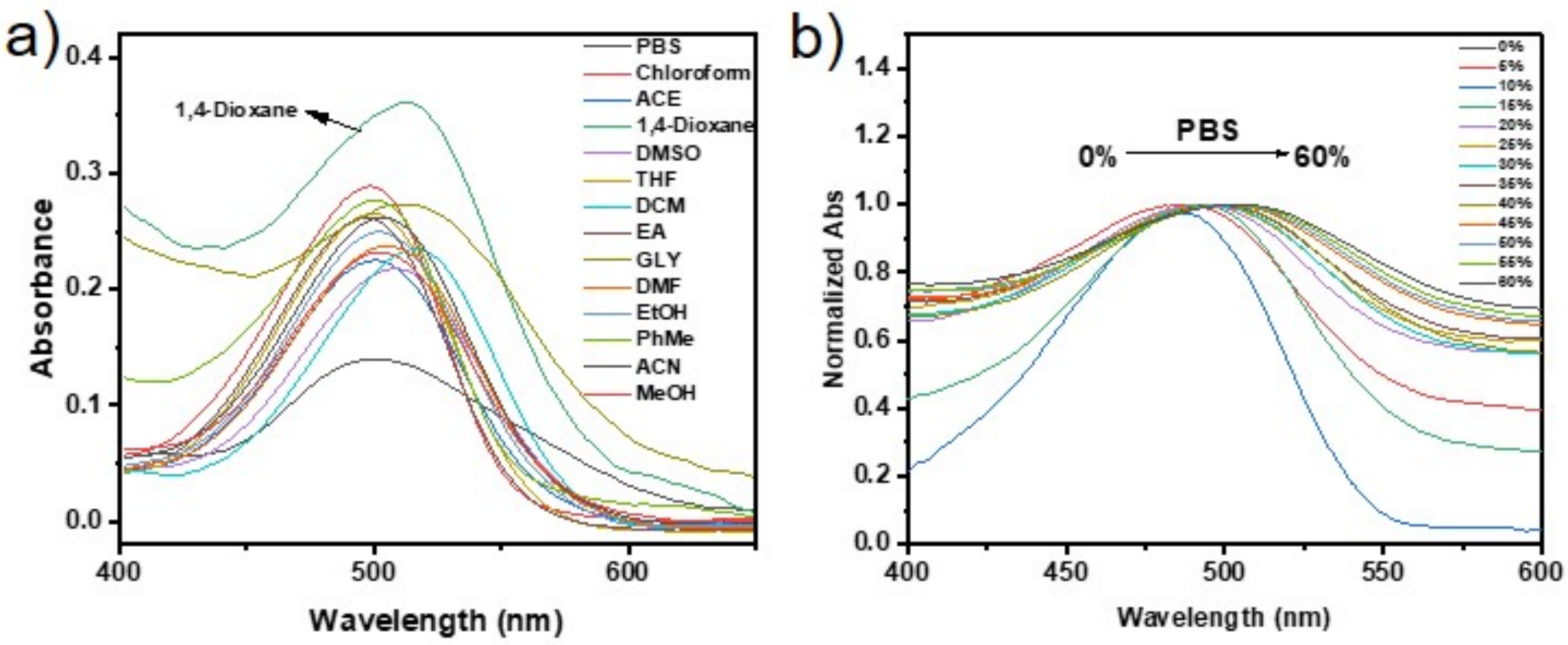

Fig. S3. (a) UV-vis absorption spectra of probe **CTAT** (10 μM) in various solvents, (b) UV spectra of **CTAT** (10 μM) in PBS/1,4-dioxane solvent mixtures with different volume ratios.

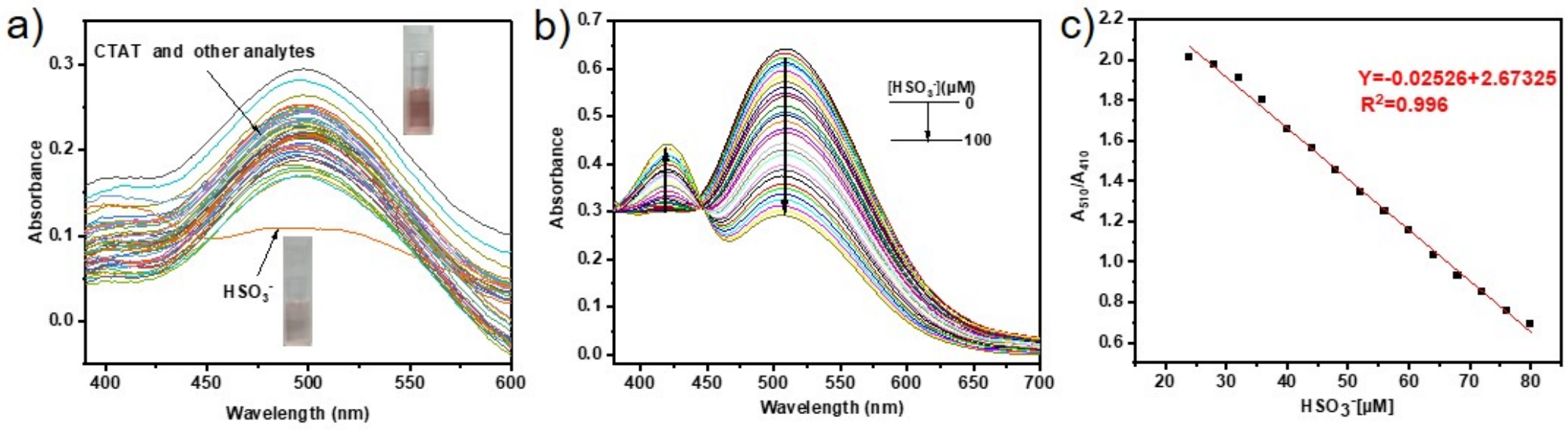

Fig. S4. (a) UV-vis absorption spectra of probe **CTAT** (10 μM) with various analytes (Inset: image of probe **CTAT** with and without HSO_3^-). (b) UV-vis absorption spectra of probe **CTAT** (10 μM) with different concentrations of HSO_3^- (0–100 μM). (c) The linear relationship of the absorbance intensity ratio (A_{510}/A_{410}) of probe **CTAT** on HSO_3^- concentration.

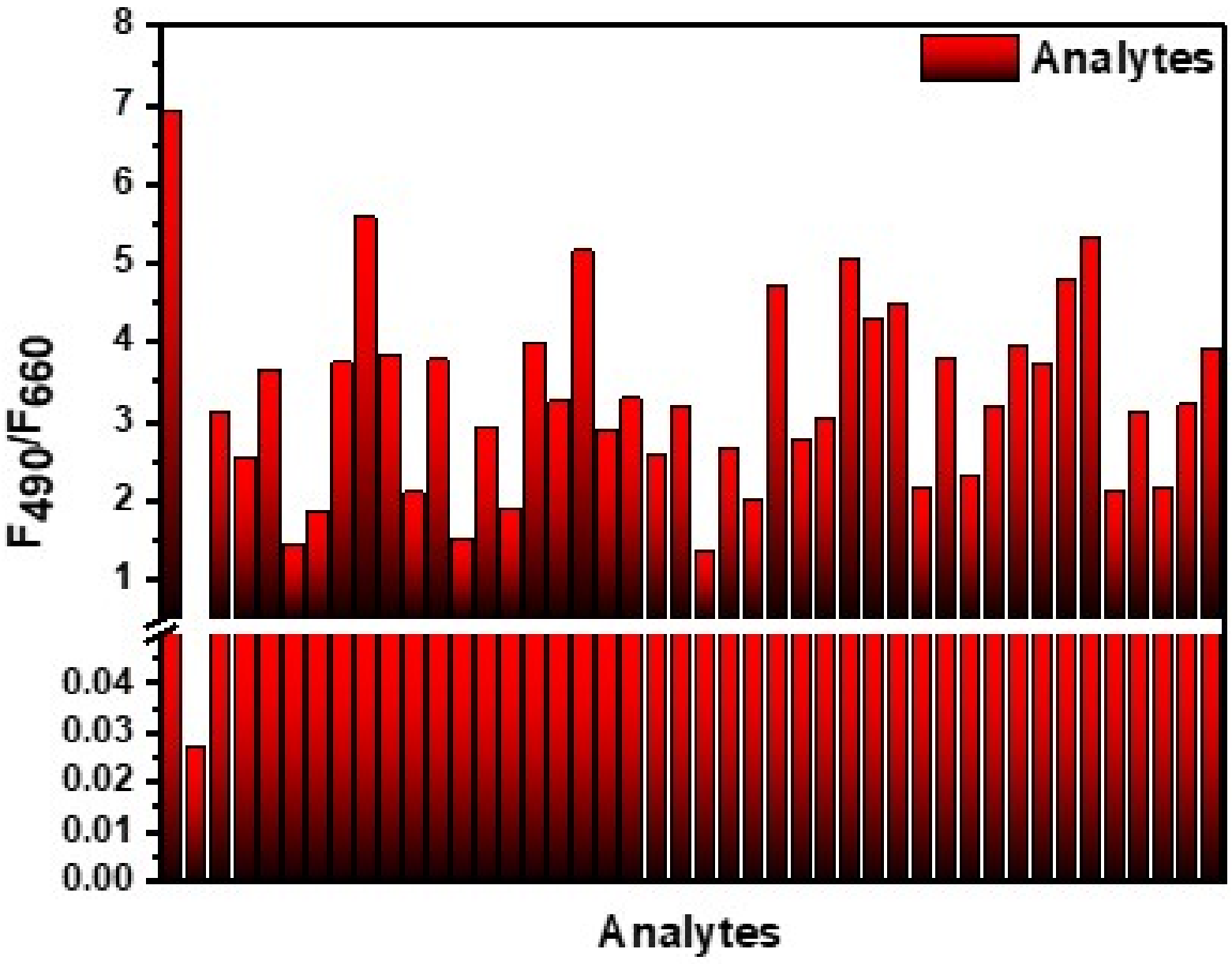

Fig. S5. The ratio (F_{465}/F_{545}) value of **CTAT** (10 μM) to HSO_3^- co-existence of various competing analytes. 1.Probe; 2. None; 3. Ag^+; 4.Al^{3+}; 5. Br^-; 6. Ca^{2+}; 7. Cd^{2+}8. Cl^-; 9. ClO^-; 10. ClO_4^-; 11. Co^{3+}; 12. Cr^{3+}; 13. Cu^{2+}; 14. F^-; 15. Fe^{3+}; 16. $H_2PO_4^-$; 17. Hg^{2+}; 18. HPO_4^{2-}; 19. HSO_4^-; 20. SO_3^{2-}; 21. SO_4^{2-}; 22. Mg^{2+}; 23. Mn^{2+}; 24 Na^+; 25. Ni^{2+}; 26. OAc^-; 277. Pb^{2+}; 28. PO_4^{3-}; 29. PPi; 30. Zn^{2+}; 31. I^-; 32. K^+; 33. $S_2O_5^{2-}$; 34. $S_2O_4^{2-}$; 35. $S_2O_3^{2-}$; 36. Hcy; 37. Cys; 38. S^{2-}; 39. GSH; 40. $^1O_2$; 41. Ala; 42. NO·; 43. Leu. 44. ROO·.

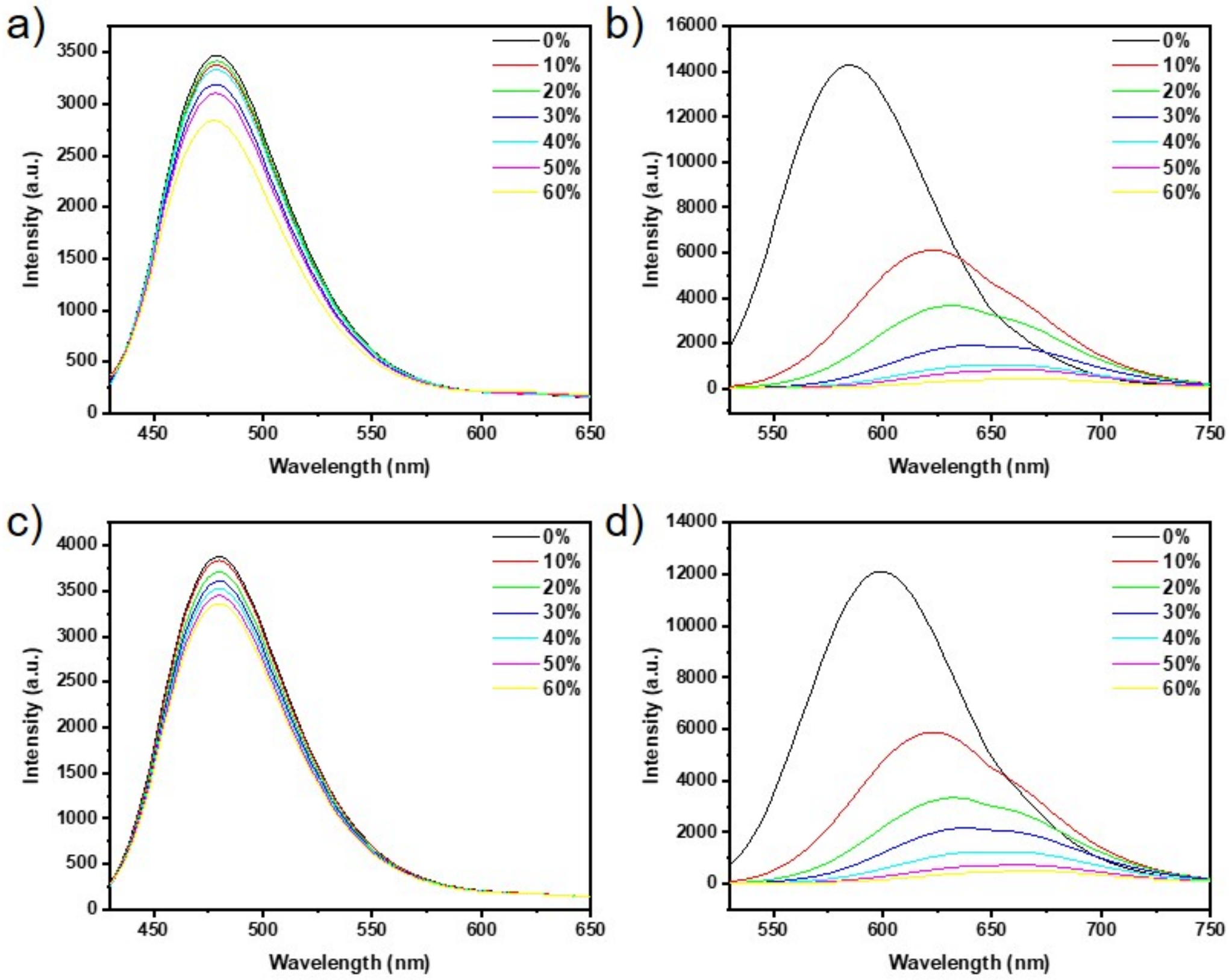

Fig. S6. Spectroscopic evaluation of the crosstalk between SO_2 derivative sensing and polarity detection by CTAT. The fluorescence response of CTAT toward SO_2 derivatives (a) was measured under different polarity conditions (b), while the polarity-dependent fluorescence response (d) was evaluated in the presence of SO_2 derivatives (c).

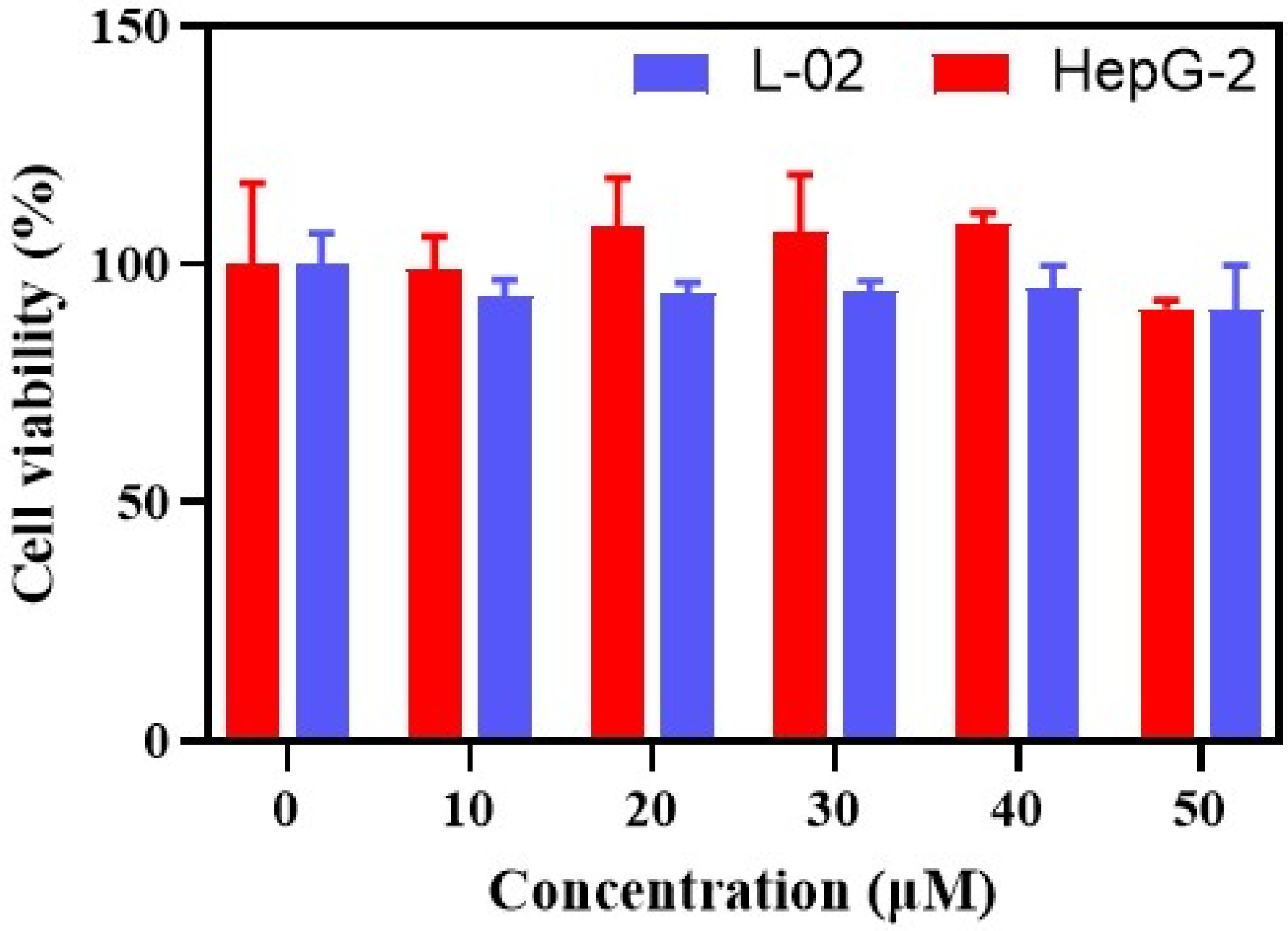

Fig. S7. CCK-8 method for estimating cell viability of L-02 and HepG2 cells, treated with various on concentrations of **CTAT**.

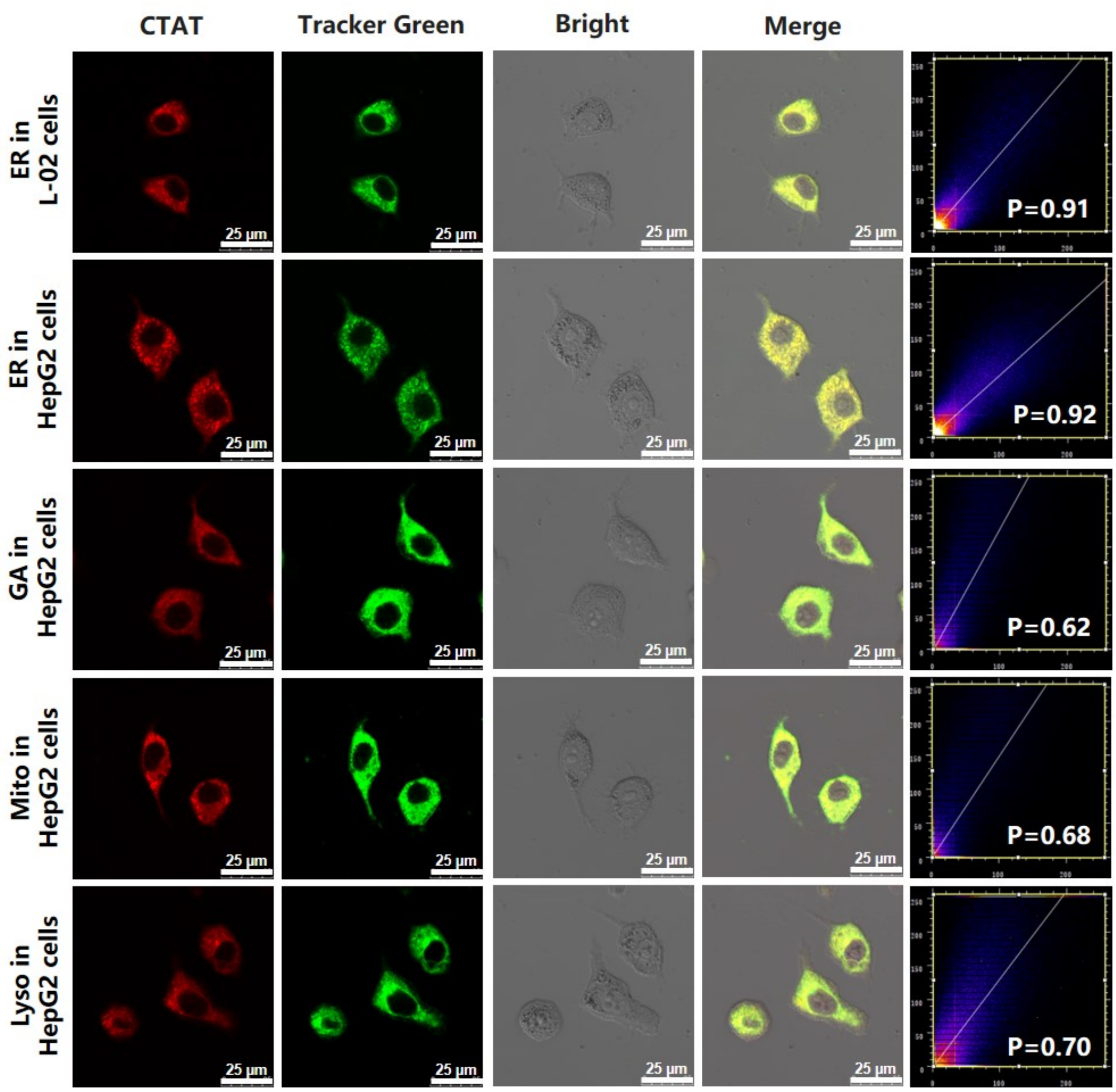

Fig. S8. Co-localization fluorescence imaging of **CTAT** and ER Tracker-Green in HepG2 cells. Scale bar is 25 μm. Colocalization imaging of **CTAT** with different organelle trackers in cells. The L-02 cells were co-stained with **CTAT** and ER-Tracker Green (first row). The HepG2 cells were co-stained with **CTAT**, and commercial dyes including ER-Tracker Green (second row), Golgi-Tracker Green (third row), Mito-Tracker Green (fourth row) and Lyso-Tracker Green (fifth row). Pearson 's colocalization correlation of **CTAT** with other organelle dyes (fifth column). Scale bar = 25 μm.

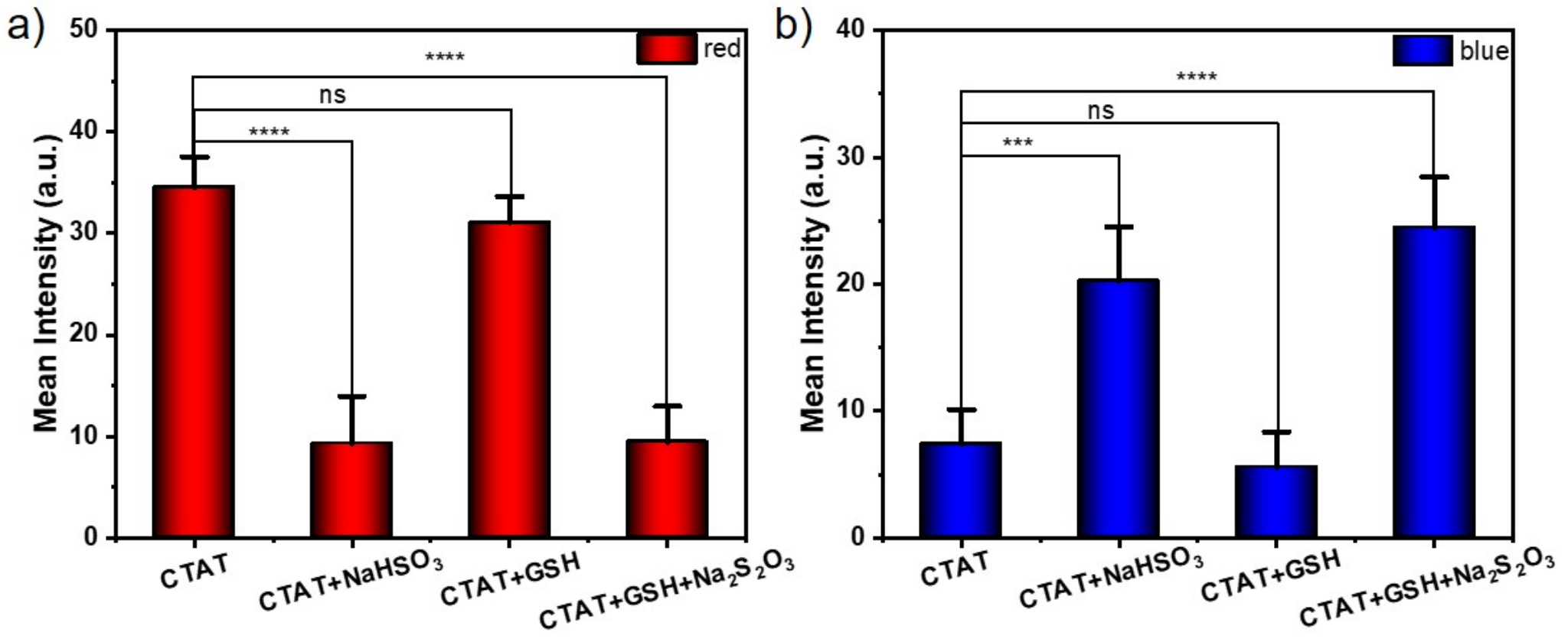

Fig. S9. Mean fluorescence intensity of the HSO_3^- levels with **CTAT** inside cultured HepG2 cells in red channel (a) and blue channel (b). Data were assessed as mean ± SD (n = 6, ***P < 0.001, ****P < 0.0001).

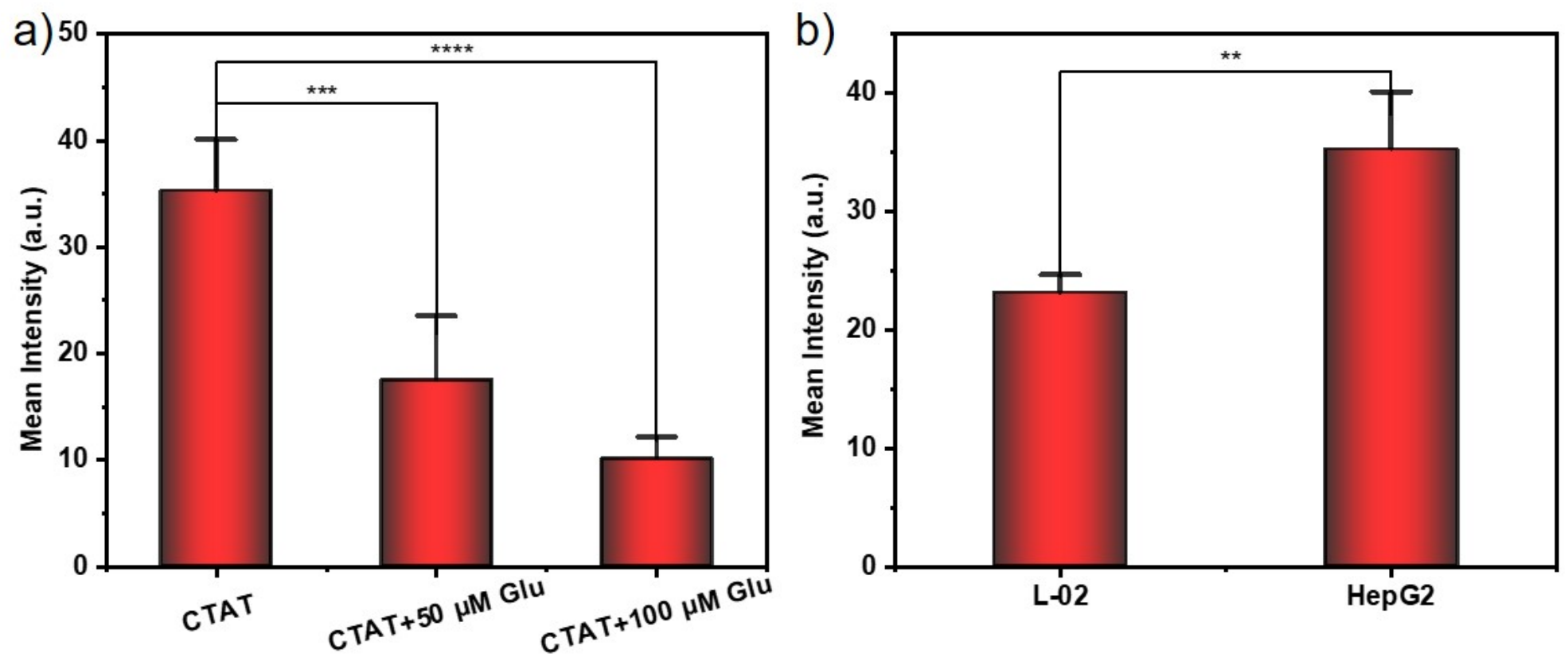

Fig. S10. Mean fluorescence intensity of **CTAT** for monitoring intracellular polarity inside cultured HepG2 cells and L-02 cells in red channel. Data were assessed as mean ± SD (n = 6, **P < 0.01, ***P < 0.001, ****P < 0.0001).

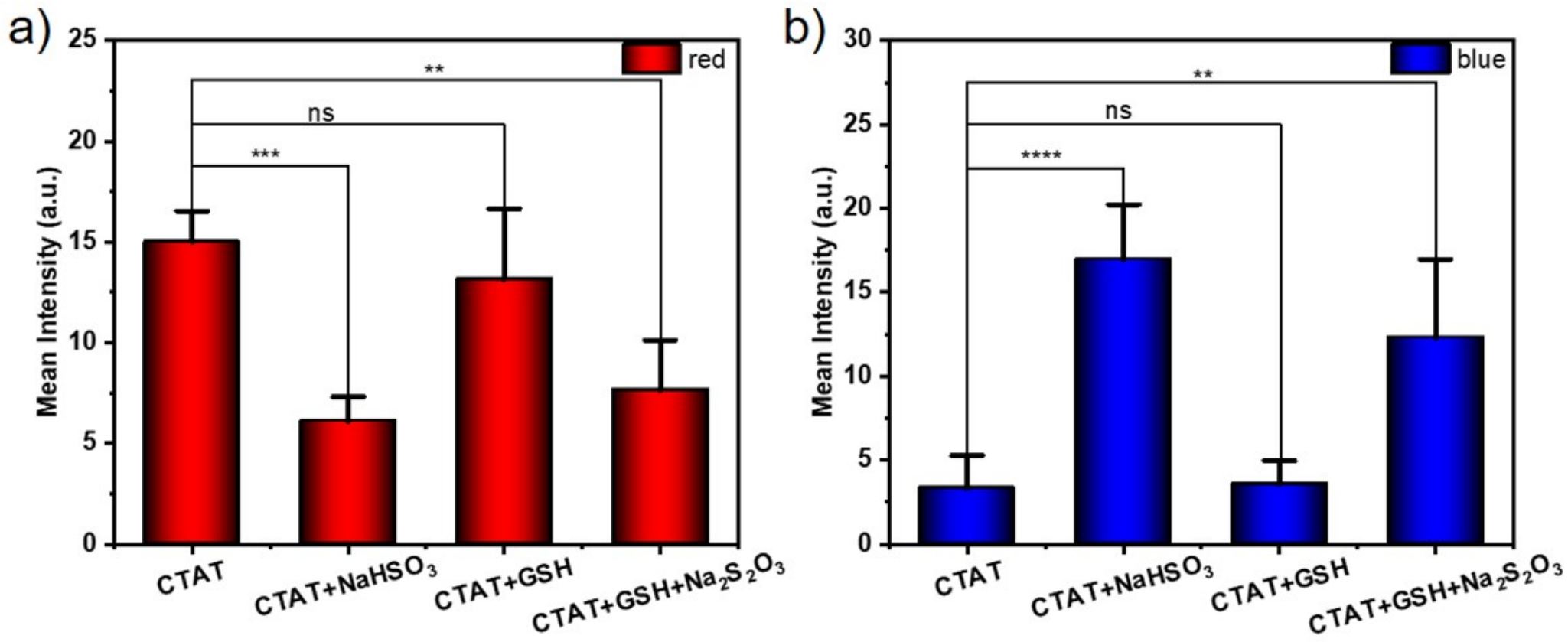

Fig. S11. Mean fluorescence intensity of the HSO_3^- levels with **CTAT** inside cultured zebrafish in red channel (a) and blue channel (b). Data were assessed as mean ± SD (n = 6, **P < 0.01, ***P < 0.001, ****P < 0.0001).

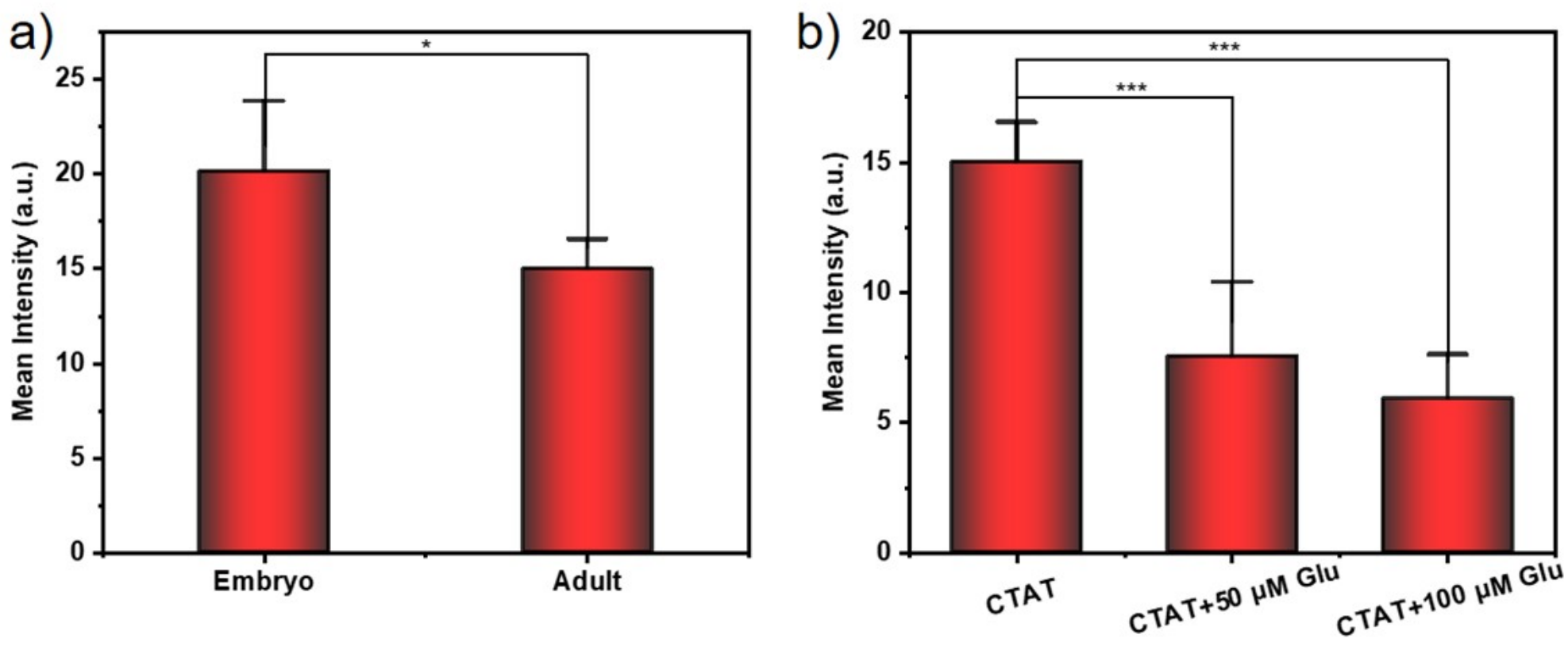

Fig. S12. Mean fluorescence intensity of **CTAT** for monitoring intracellular polarity inside cultured embryo and adult fish in red channel. Data were assessed as mean ± SD (n = 6, *P < 0.1, ***P < 0.001, ****P < 0.0001).

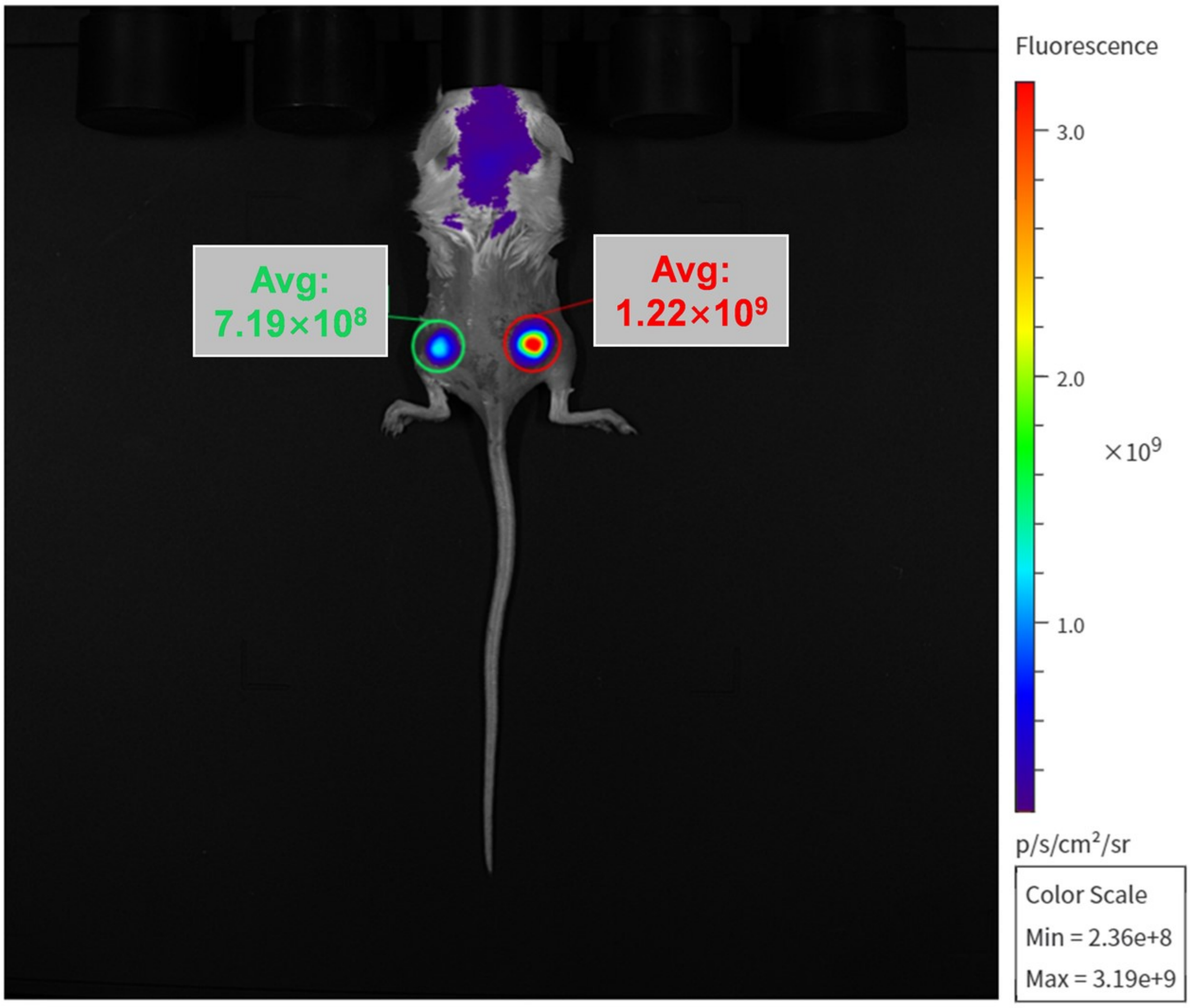

Fig. S13. Fluorescence imaging of normal and tumor tissues of tumor-bearing after subcutaneous injection of **CTAT** (red circle indicates the tumor area, greed circle indicates the normal area).

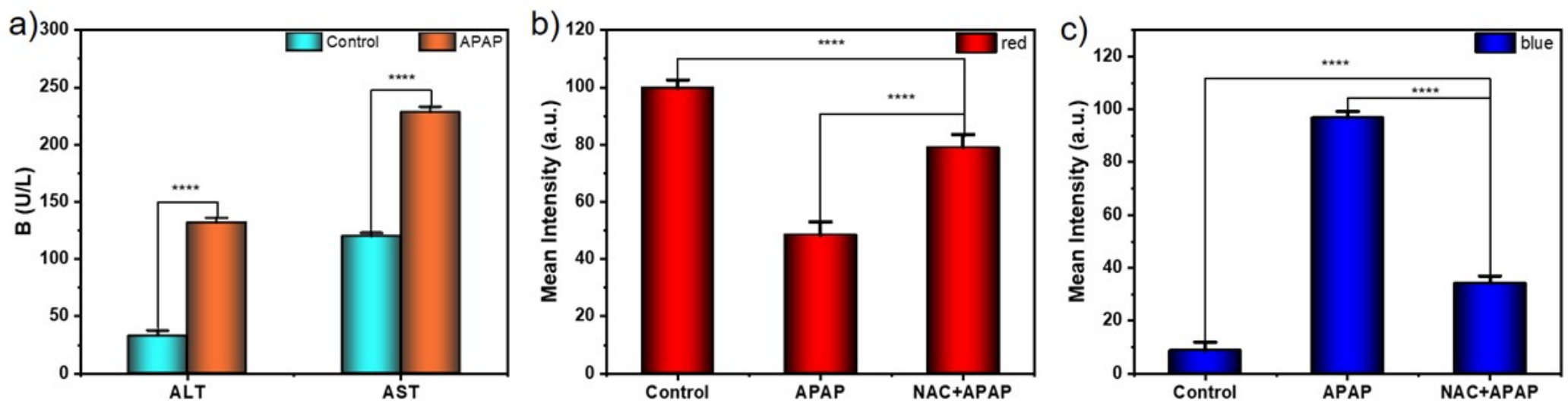

Fig. S14. (a) Concentration of ALT and ALS in the control group and APAP group. Mean fluorescence intensity of the control, APAP and APAP+NAC groups in red channel (b) and blue channel (c). Data were assessed as mean ± SD (n = 6, ****P < 0.0001).

3. References:

1. Peng, Z.; Shi, L.; Zeng, X.; Yang, S.; Xiao, J.; Gong, S.; Xiang, H.; Shao, G., Rational design of the ratiometric fluorescent probes for sulfur dioxide derivatives and the study on the sensing performance of cyano-containing groups. *Dyes and Pigments* **2021,** *186*.
2. Pérez, C.; Díaz-García, C. V.; Agudo-López, A.; del Solar, V.; Cabrera, S.; Agulló-Ortuño, M. T.; Navarro-Ranninger, C.; Alemán, J.; López-Martín, J. A., Evaluation of novel trans-sulfonamide platinum complexes against tumor cell lines. *European Journal of Medicinal Chemistry* **2014,** *76*, 360-368.
3. Desantis, J.; Nannetti, G.; Massari, S.; Barreca, M. L.; Manfroni, G.; Cecchetti, V.; Palù, G.; Goracci, L.; Loregian, A.; Tabarrini, O., Exploring the cycloheptathiophene-3-carboxamide scaffold to disrupt the interactions of the influenza polymerase subunits and obtain potent anti-influenza activity. *European Journal of Medicinal Chemistry* **2017,** *138*, 128-139.
